# Catalytic two-electron reduction of dioxygen catalysed by metal-free [14]triphyrin(2.1.1)†
†Electronic supplementary information (ESI) available: Spectroscopic, kinetic and DFT data. See DOI: 10.1039/c5sc02465j


**DOI:** 10.1039/c5sc02465j

**Published:** 2015-08-03

**Authors:** Kentaro Mase, Kei Ohkubo, Zhaoli Xue, Hiroko Yamada, Shunichi Fukuzumi

**Affiliations:** a Department of Material and Life Science , Graduate School of Engineering , ALCA and SENTAN , Japan Science and Technology Agency (JST) , Osaka University , Suita , Osaka 565-0871 , Japan . Email: fukuzumi@chem.eng.osaka-u.ac.jp; b Department of Chemistry and Nano Science , Ewha Womans University , Seoul 120-750 , Korea; c Graduate School of Materials Science , Nara Institute of Science and Technology , CREST , Japan Science and Technology Agency (JST) , Ikoma , Nara 630-0192 , Japan . Email: hyamada@ms.naist.jp; d Faculty of Science and Engineering , ALCA , SENTAN , Japan Science and Technology Agency (JST) , Meijo University , Nagoya , Aichi 468-0073 , Japan

## Abstract

The catalytic two-electron reduction of dioxygen (O_2_) by octamethylferrocene (Me_8_Fc) occurs with a metal-free triphyrin (HTrip) in the presence of perchloric acid (HClO_4_) in benzonitrile (PhCN) at 298 K to yield Me_8_Fc^+^ and H_2_O_2_. Detailed kinetic analysis has revealed that the catalytic two-electron reduction of O_2_ by Me_8_Fc with HTrip proceeds *via* proton-coupled electron transfer from Me_8_Fc to HTrip to produce H_3_Trip˙^+^, followed by a second electron transfer from Me_8_Fc to H_3_Trip˙^+^ to produce H_3_Trip, which is oxidized by O_2_*via* formation of the H_3_Trip/O_2_ complex to yield H_2_O_2_. The rate-determining step in the catalytic cycle is hydrogen atom transfer from H_3_Trip to O_2_ in the H_3_Trip/O_2_ complex to produce the radical pair (H_3_Trip˙^+^ HO_2_˙) as an intermediate, which was detected as a triplet EPR signal with fine-structure by the EPR measurements at low temperature. The distance between the two unpaired electrons in the radical pair was determined to be 4.9 Å from the zero-field splitting constant (*D*).

## Abstract 2

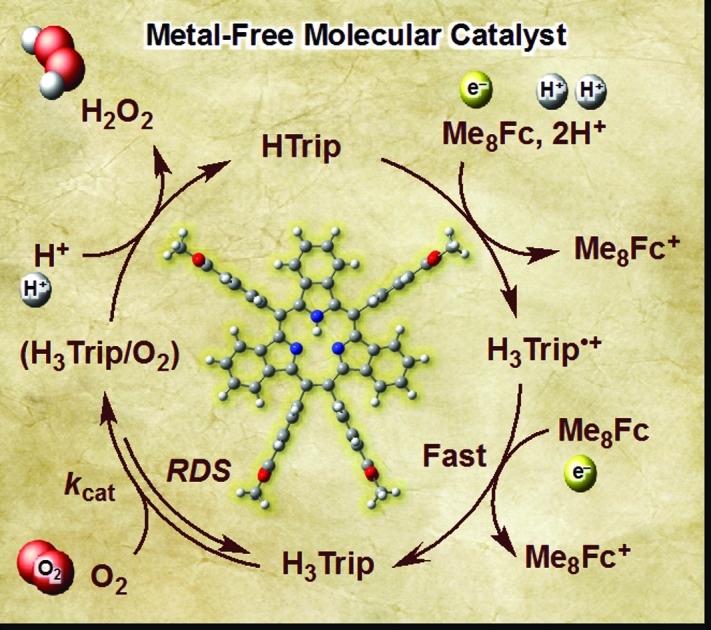
The catalytic two-electron reduction of dioxygen (O_2_) by octamethylferrocene (Me_8_Fc) has been studied by detailed kinetic analysis. This study provides valuable insight into the catalytic mechanism of the two-electron reduction of O_2_ with an organic catalyst.

## Introduction

Utilization of natural energy to produce chemical energy consisting of earth-abundant elements is an essential technology for building a society based on the sustainable use of materials. Hydrogen peroxide (H_2_O_2_) produced by two-electron reduction of O_2_ is a versatile and environmentally benign oxidant, which is widely used on a large industrial scale.^1^,^2^ Furthermore, H_2_O_2_ has been proposed as a sustainable energy carrier that can be used in fuel cells, where direct and efficient conversion of chemical to electrical energy is required.^3^–^5^ However, the anthraquinone process, currently used to produce H_2_O_2_ in industry, requires potentially explosive hydrogen and a noble metal catalyst.^6^ Extensive efforts have so far been devoted to provide an alternative way to produce H_2_O_2_ photochemically or thermally without the use of noble metal catalysts.^7^–^13^ In many cases, redox-active transition metal-based complexes such as cobalt,^14^–^23^ iron,^24^–^27^ and copper complexes,^28^–^31^ have been employed as O_2_ reduction catalysts, because triplet O_2_ is inactive towards organic compounds due to spin restriction in the absence of an appropriate catalyst.^32^

Recently, nitrogen-doped carbon materials have attracted increasing attention as an efficient metal-free catalyst for the catalytic reduction of O_2_.^33^–^35^ However, the catalytic mechanism has yet to be well understood, because few spectroscopic studies to detect reaction intermediates in a catalytic cycle have been performed on heterogeneous systems. In homogeneous systems, reduced flavin analogues involved in flavoenzymes have so far been known to play a pivotal role in the catalytic reduction of O_2_, which is a key step of biological oxidation.^36^,^37^ In particular, the deprotonated states of reduced flavin analogues, which are thermodynamically more able to reduce O_2_*via* an electron-transfer process, are considered to be a reactive intermediate in the reduction of O_2_.^38^

On the other hand, Girault and coworkers recently reported that the free base porphyrin has the ability to catalyse the two-electron reduction of O_2_ using one-electron reductants such as ferrocene at liquid–liquid interfaces.^39^ In such systems, although the catalytic mechanism of metal-free organocatalysts has yet to be clarified, the oxidation state of the organocatalyst is thought to remain the same during the catalytic reduction of O_2_. Thus, no electron-transfer reduction of organic catalysts has been reported in relation to the catalytic reduction of O_2_.

In this context, Nocera and coworkers recently reported the stabilization of the peroxide dianion within the cavity of a hexacarboxamide cryptand,^40^ where strong hydrogen bond donors are arranged to completely surround the peroxide dianion with a partial positive charge. This result provides support for the proposal that metal-free organocatalysts, which have multiple hydrogen bonding moieties, can efficiently catalyse O_2_ reduction.

**Chart 1:**
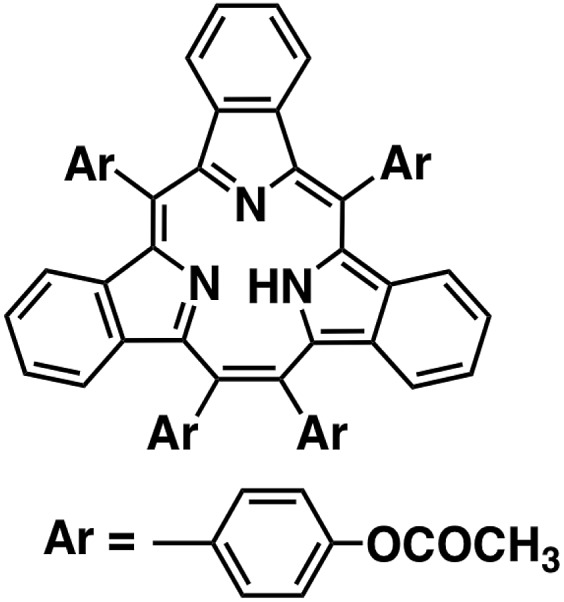
Structure of HTrip.

We report herein the catalytic two-electron reduction of O_2_ by an one-electron reductant, octamethylferrocene (Me_8_Fc), with metal-free [14]triphyrin(2.1.1) (denoted as HTrip in Chart 1)^41^ in the presence of HClO_4_ in benzonitrile (PhCN) at 298 K. The catalytic mechanism for the O_2_ reduction by Me_8_Fc is clarified on the basis of a detailed kinetic study. Proton-coupled electron-transfer reduction of HTrip by Me_8_Fc results in the formation of the reduced state of HTrip, and this resulting reduced HTrip is oxidized by O_2_ to reproduce HTrip, indicating that HTrip acts as a metal-free catalyst for the reduction of O_2_ by Me_8_Fc in the presence of HClO_4_ in PhCN. This discovery of a reactive intermediate in the catalytic O_2_ reduction with a molecular organic catalyst provides valuable insight into the development of an efficient metal-free catalyst for the reduction of O_2_.

## Results and discussion

### Protonation of HTrip with HClO_4_

**Fig. 1:**
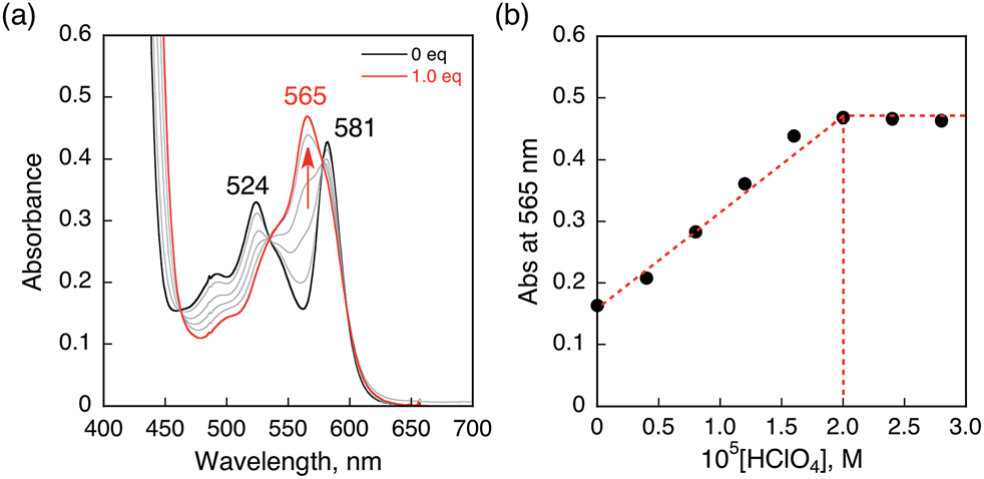
(a) Absorption spectral changes of HTrip (2.0 × 10^–5^ M) upon the addition of HClO_4_ in air-saturated PhCN at 298 K. (b) Absorbance change profile at 565 nm.

HTrip was protonated by addition of perchloric acid (HClO_4_) to an air-saturated benzonitrile (PhCN) solution of HTrip. The characteristic absorption bands for HTrip at 524 and 581 nm decreased in intensity, with an increase in the absorption band at 565 nm, exhibiting clean isosbestic points, as shown in Fig. 1a. As can be seen in Fig. 1b, the absorbance change at 565 nm is saturated in the presence of 1 equiv. of HClO_4_. Thus, HTrip is protonated to afford H_2_Trip^+^, as given by eqn (1).

1 HTrip + H^+^ → H_2_Trip^+^

The p*K*_a_ value of H_2_Trip^+^ in PhCN was estimated to be 15.6 from the titration of HTrip with trifluoroacetic acid (TFA), as shown in Fig. S1 in the ESI.† The p*K*_a_ value of H_2_Trip^+^ is slightly larger than that of free base porphyrin analogues.^42^ There is no further protonation due to strong repulsion between NH protons in the small macrocyclic ligand, as reported previously.^41^

### Electrochemical measurements of HTrip in the presence of HClO_4_

**Fig. 2:**
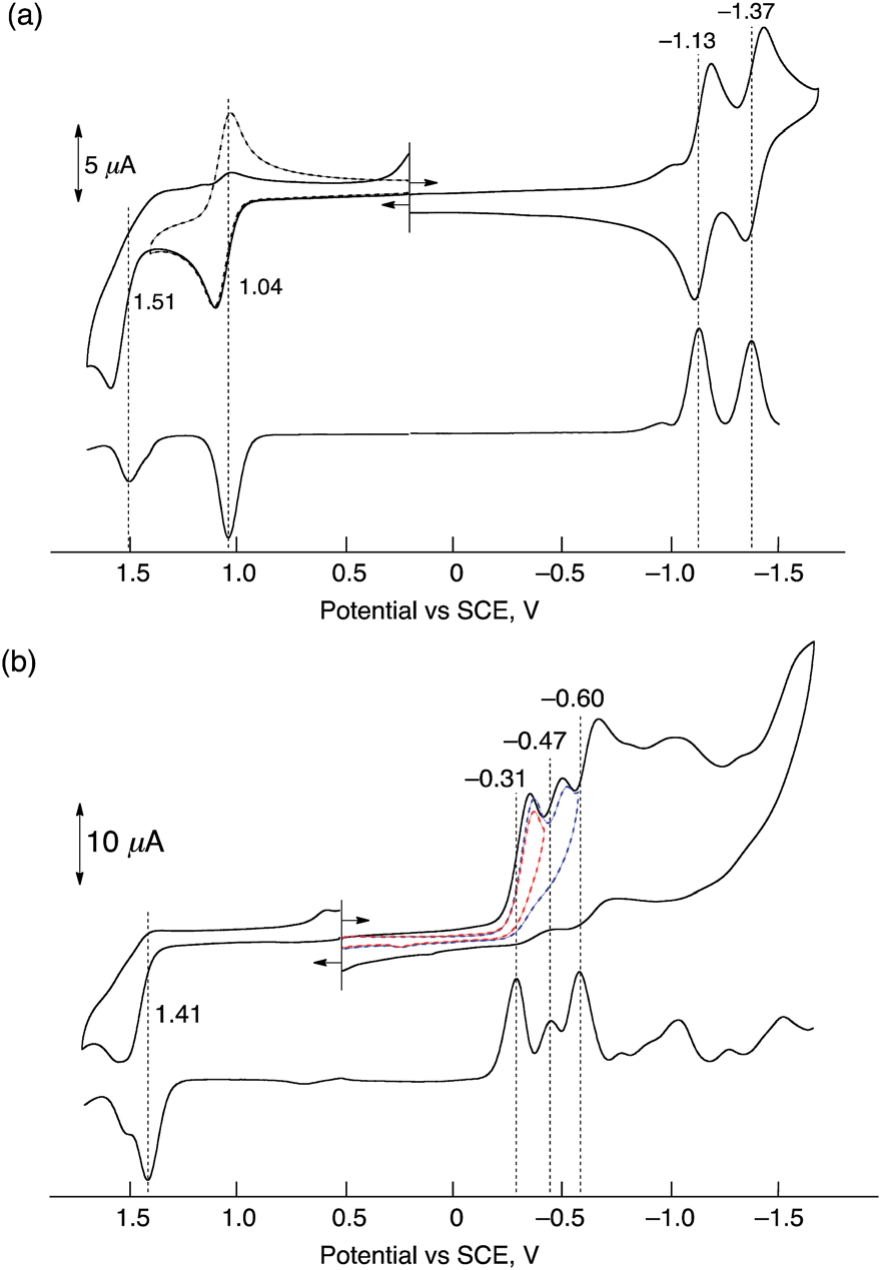
Cyclic voltammograms (upper) and differential pulse voltammograms (lower) of deaerated PhCN solutions of HTrip (1.0 × 10^–3^ M) recorded in the presence of TBAPF_6_ (0.10 M) (a) without HClO_4_ and (b) with HClO_4_ (1.0 × 10^–2^ M); sweep rate: 100 mV s^–1^ for CV and 4 mV s^–1^ for DPV.

Electrochemical measurements of HTrip were performed in deaerated PhCN containing 0.10 M TBAPF_6_, as shown in Fig. 2. A cyclic voltammogram of HTrip exhibits reversible reduction waves at *E*_1/2_ = –1.13 and –1.37 V (*vs.* SCE), which correspond to the first and second one-electron reduction of HTrip. The first one-electron oxidation occurs at *E*_1/2_ = 1.04 V, which is followed by an irreversible oxidation (Fig. 2a). The formation of HTrip˙^–^ was detected by UV-vis absorption spectra in the electrochemical reduction of HTrip at a controlled potential of –1.25 V *vs.* SCE in the thin-layer cell, as shown in Fig. S2 in the ESI.† By addition of HClO_4_, the first reduction potential of HTrip was positively shifted from *E*_1/2_ = –1.13 V to –0.31 V (*vs.* SCE) because of the protonation of HTrip, but the reduction became irreversible (Fig. 2b). In such a case, proton-coupled electron transfer from an electron donor with the one-electron oxidation potential, which is less negative than –0.31 V, to HTrip may be thermodynamically feasible (*vide infra*).

### Electron-transfer reduction of HTrip in the presence of HClO_4_

**Scheme 1:**
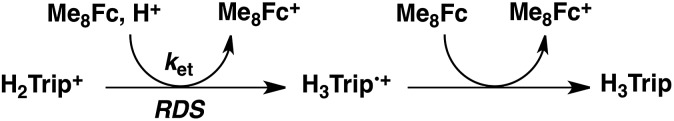


**Fig. 3:**
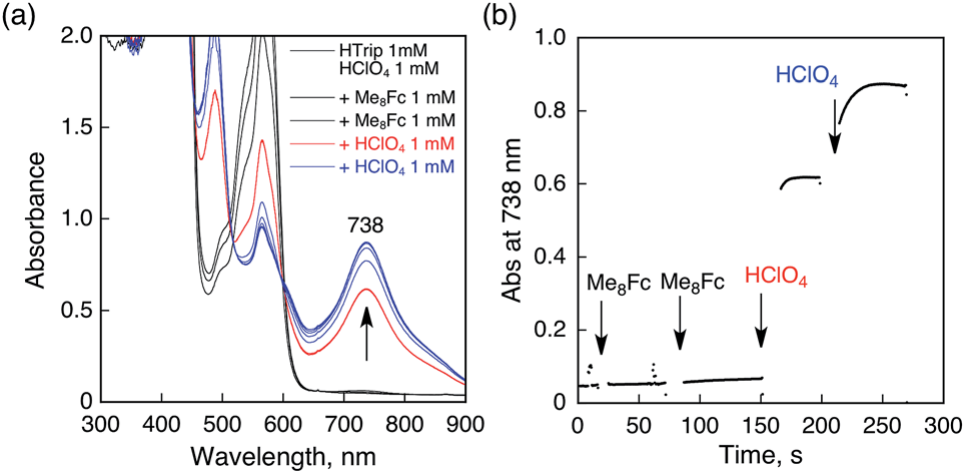
(a) Absorption spectral changes upon addition of Me_8_Fc (1.0 × 10^–3^ and 2.0 × 10^–3^ M) to a deaerated PhCN solution of H_2_Trip^+^ (1.0 × 10^–3^ M) in the presence of HClO_4_ (1.0 × 10^–3^ M) at 298 K in a quartz cuvette (light path length = 1 mm) (black); absorption spectral change upon addition of HClO_4_ (1.0 × 10^–3^ M) to the solution indicated by the black line (red); absorption spectral change upon addition of HClO_4_ (1.0 × 10^–3^ M) to the solution indicated by the red line (blue). (b) Absorption change at 738 nm upon addition of various concentrations of Me_8_Fc and HClO_4_.

No electron transfer from Me_8_Fc to HTrip occurred in the absence of HClO_4_ in PhCN at 298 K, as indicated by the more negative *E*_1/2_ value of HTrip (–1.13 V *vs.* SCE) as compared with that of Me_8_Fc (–0.04 V *vs.* SCE).^8^ However, the addition of more than two equiv. of HClO_4_ to a deaerated PhCN solution of Me_8_Fc and HTrip resulted in the appearance of an absorption band at 738 nm due to H_3_Trip with clean isosbestic points, as shown in Fig. 3. It should be noted that no electron transfer from Me_8_Fc to H_2_Trip^+^ occurred in the presence of one equiv. of HClO_4_, as shown in Fig. 3b. These results indicate that uphill electron transfer from Me_8_Fc to H_2_Trip^+^ is coupled with protonation of H_2_Trip˙ to produce H_3_Trip˙^+^, followed by fast electron transfer from Me_8_Fc to H_3_Trip˙^+^ to yield H_3_Trip. Thus, the second protonation in fact occurs by coupling with reduction of H_2_Trip^+^ (*i.e.* H_3_Trip˙^+^ is accessible but not H_3_Trip^2+^). The stoichiometry of the overall reaction is given in Scheme 1.

**Fig. 4:**
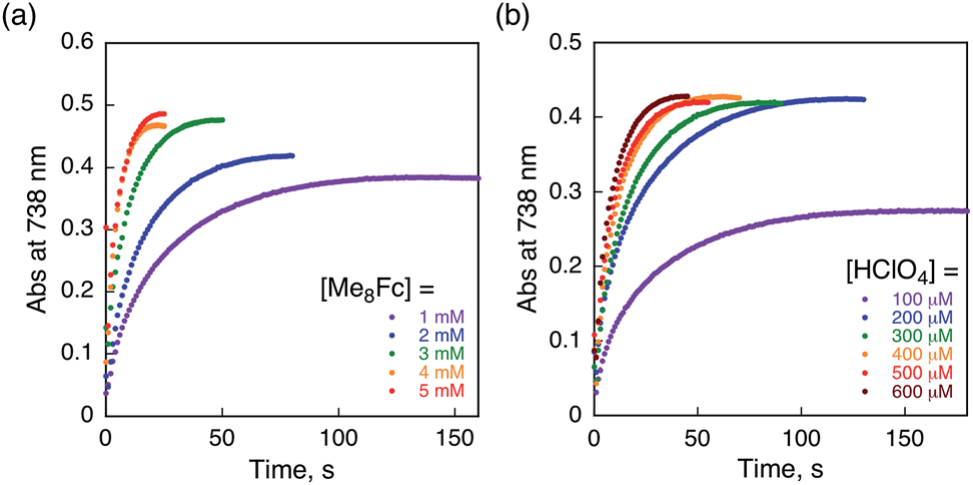
Time profiles of absorbance at 738 nm due to H_3_Trip in the reduction of H_2_Trip^+^ (2.5 × 10^–5^ M) (a) by various concentrations of Me_8_Fc in the presence of HClO_4_ (3.0 × 10^–4^ M) and (b) by Me_8_Fc (2.0 × 10^–3^ M) in the presence of various concentrations of HClO_4_ in deaerated PhCN at 298 K.

**Fig. 5:**
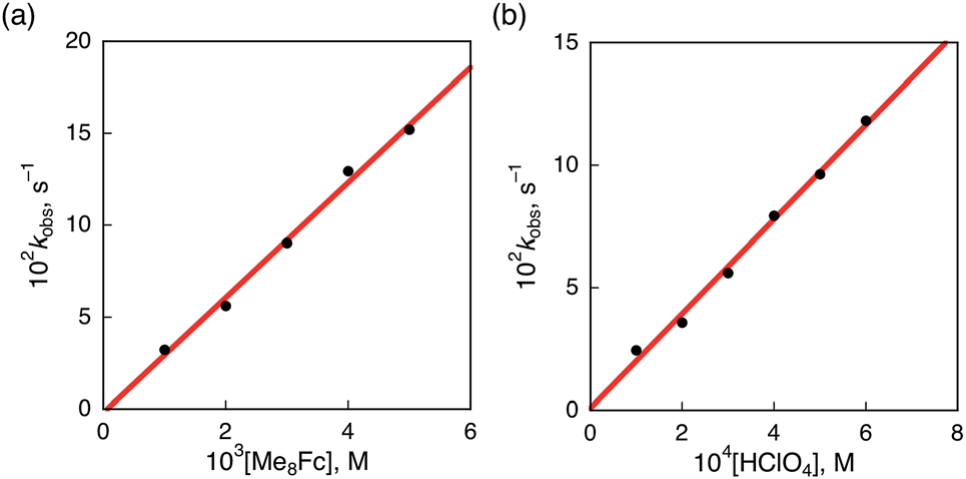
(a) Plot of *k*_obs_*vs.* [Me_8_Fc] for the reduction of H_2_Trip^+^ (2.5 × 10^–5^ M) by various concentrations of Me_8_Fc in the presence of HClO_4_ (3.0 × 10^–4^ M) in PhCN at 298 K. (b) Plot of *k*_obs_*vs.* [HClO_4_] for the reduction of H_2_Trip^+^ (2.5 × 10^–5^ M) by Me_8_Fc (2.0 × 10^–3^ M) in the presence of various concentrations of HClO_4_ in deaerated PhCN at 298 K.

The rate of proton-coupled electron-transfer reduction of H_2_Trip^+^ (*k*_et_) to form H_3_Trip˙^+^ was determined from the dependence of the observed rate constant (*k*_obs_) on concentrations of Me_8_Fc and HClO_4_, as shown in Fig. 4. The *k*_obs_ value was determined from the increase in absorbance at 738 nm due to H_3_Trip, which obeyed first-order kinetics (Fig. S3 in the ESI†). The *k*_obs_ value increased linearly with increasing concentrations of Me_8_Fc and HClO_4_, as shown in Fig. 5. Thus, the rate of formation of H_3_Trip is given by eqn (2).

2 d[H_3_Trip]/d*t* = *k*_et_[H_2_Trip^+^][HClO_4_][Me_8_Fc]

The *k*_et_ value is determined from the slope of the linear plot of *k*_obs_*vs.* [Me_8_Fc] and [HClO_4_] to be (9.8 ± 0.2) × 10^4^ M^–2^ s^–1^. The *k*_et_ value of the proton-coupled electron-transfer reduction of H_2_Trip^+^ by Me_10_Fc was also determined from the slope of the linear plot of *k*_obs_*vs.* [Me_10_Fc] and [HClO_4_] to be (3.1 ± 0.3) × 10^5^ M^–2^ s^–1^ (Fig. S4–S6 in the ESI†). The *k*_et_ value for Me_10_Fc is larger than that for Me_8_Fc because Me_10_Fc (*E*_ox_ = –0.08 V *vs.* SCE) is a stronger electron donor than Me_8_Fc (–0.04 V *vs.* SCE).^28^

The formation of H_3_Trip was also confirmed by the electrochemical reduction of H_2_Trip^+^ monitored by the UV-vis spectral change at an applied potential of –0.30 V *vs.* SCE in the thin-layer cell, as shown in Fig. S7 (in the ESI†). The product obtained after the electrochemical reduction of H_2_Trip^+^ at –0.30 V displayed the characteristic absorption band at 738 nm. The same absorption band was seen in the chemical reduction of H_2_Trip^+^ by Me_8_Fc in the presence of HClO_4_ (Fig. 2).

**Scheme 2:**
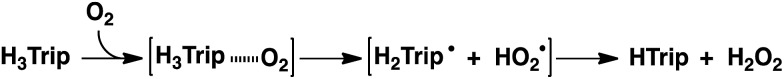


**Fig. 6:**
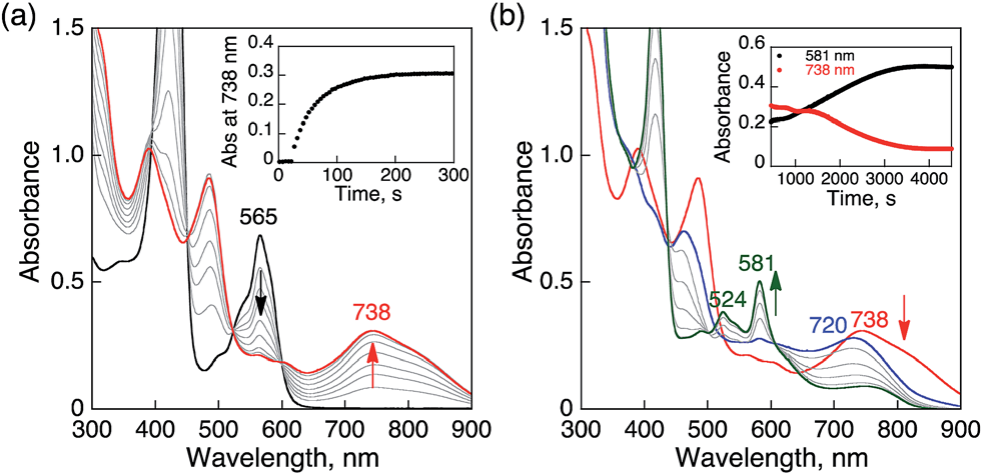
(a) Absorption spectral changes produced by electron transfer from Me_8_Fc (1.0 × 10^–4^ M) to HTrip (2.5 × 10^–5^ M) in the presence of HClO_4_ (1.0 × 10^–4^ M) in deaerated PhCN at 298 K. (b) Absorption spectral changes upon introducing O_2_ to a deaerated PhCN solution of (a). The red and green lines show the spectrum of H_3_Trip before and after introducing O_2_ by O_2_ gas bubbling, respectively. The blue line shows the spectrum due to precursor complex. Insets show absorption time profiles.

When O_2_ was introduced to a deaerated PhCN solution of H_3_Trip produced by the proton-coupled electron transfer from Me_8_Fc to HTrip in the presence of HClO_4_, the absorption band at 738 nm due to H_3_Trip was immediately changed to a new absorption band at 720 nm, which can be attributed to the formation of the O_2_ complex, as shown in Scheme 2 (*vide infra*). Subsequently, this spectrum decreased gradually, accompanied by the regeneration of HTrip as shown in Fig. 6. This indicates that H_3_Trip was readily oxidized by O_2_ to produce HTrip and H_2_O_2_ (Scheme 2).

### Catalytic two-electron reduction of O_2_ by Me_8_Fc with HTrip in the presence of HClO_4_

**Fig. 7:**
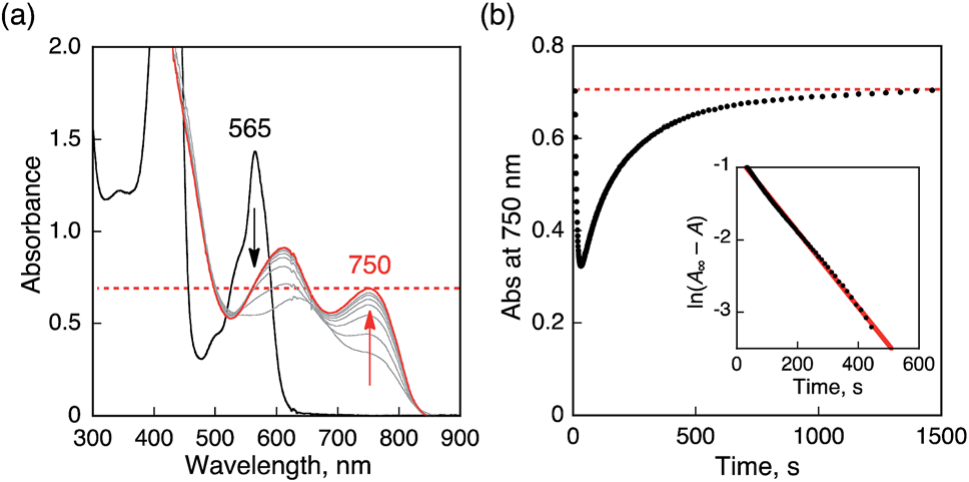
(a) Absorption spectral changes in the two-electron reduction of O_2_ (9.4 × 10^–4^ M) by Me_8_Fc (1.0 × 10^–2^ M) with HTrip (5.0 × 10^–5^ M) in the presence of HClO_4_ (1.0 × 10^–2^ M) in PhCN at 298 K. The black and red lines show the spectra before and after addition of Me_8_Fc, respectively. The dotted line is the absorbance at 750 nm due to 1.9 × 10^–3^ M of Me_8_Fc^+^. (b) Time profile of absorbance at 750 nm due to Me_8_Fc^+^. Inset shows first-order plot.

The proton-coupled electron-transfer reduction of HTrip by Me_8_Fc (Scheme 1) and the oxidation of the resulting reduced HTrip (H_3_Trip) by O_2_ (Scheme 2) indicate that HTrip acts as a metal-free catalyst for the reduction of O_2_ by Me_8_Fc in the presence of HClO_4_ in PhCN. Indeed, the addition of Me_8_Fc to air-saturated PhCN at 298 K containing a catalytic amount of HTrip and a large excess of HClO_4_ resulted in the efficient oxidation of Me_8_Fc by O_2_ to yield Me_8_Fc^+^, as shown in Fig. 7a.

The formation of Me_8_Fc^+^ was monitored by a rise in absorbance at 750 nm due to Me_8_Fc^+^ (Fig. 7b). When an excess amount of Me_8_Fc relative to O_2_ (*i.e.*, [O_2_] limiting conditions) was employed, the concentration of produced Me_8_Fc^+^ (1.9 × 10^–3^ M) was twice that of O_2_ (9.4 × 10^–4^ M). In addition, the stoichiometric production of H_2_O_2_ was confirmed by iodometric titration, as shown in Fig. S8 (in the ESI†). In contrast, when an excess amount of O_2_ relative to Me_8_Fc (*i.e.*, [Me_8_Fc] limiting conditions) was employed, the concentration of produced H_2_O_2_ (1.0 × 10^–3^ M) was half that of Me_8_Fc (2.0 × 10^–3^ M), where the amount of H_2_O_2_ was determined by the reaction with [(TMC)Fe^II^](OTf)_2_ (TMC = 1,4,8,11-tetramethyl-1,4,8,11-tetraazacyclotetradecane) to produce the corresponding Fe(iv)-oxo complex ([TMC]Fe^IV^(O))^2+^, as shown in Fig. S9 (in the ESI†).^43^ Thus, the stoichiometry of the catalytic reduction of O_2_ by Me_8_Fc has been firmly established, as given in eqn (3).3
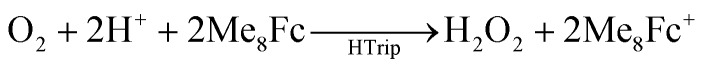



The rate of formation of Me_8_Fc^+^ in the catalytic reduction of O_2_ with excess Me_8_Fc and HClO_4_ in Fig. 7b obeys first-order kinetics. It should be noted that the oxidation of Me_8_Fc by O_2_ hardly occurred in the absence of HTrip under the present experimental conditions, as shown in Fig. S10 (in the ESI†). When Me_8_Fc was replaced by weaker one-electron reductants such as ferrocene (Fc : *E*_ox_ = 0.37 V *vs.* SCE) and dimethylferrocene (Me_2_Fc : *E*_ox_ = 0.26 V *vs.* SCE), no changes in the absorption band of H_2_Trip^+^ at 565 nm were observed, as shown in Fig. S11 (in the ESI†). When Me_8_Fc was replaced by a stronger one-electron reductant, *i.e.*, decamethylferrocene (Me_10_Fc : *E*_ox_ = –0.10 V *vs.* SCE), greatly enhanced oxidation of Me_10_Fc occurred with the decrease in absorbance at 565 nm due to H_2_Trip^+^ (Fig. S12a in the ESI†). In the case of Me_10_Fc, however, the oxidation of Me_10_Fc by O_2_ occurred without HTrip in the presence of HClO_4_ in PhCN (Fig. S12c in the ESI†). These results indicate that the reduction of H_2_Trip^+^ to produce H_3_Trip is essential in the catalytic reduction of O_2_ to produce H_2_O_2_.

When a metal complex of HTrip, *η*^5^-cyclopentadienyliron(ii) [14]triphyrin(2.1.1) (CpFe^II^Trip),41*c* was employed as an O_2_ reduction catalyst instead of HTrip for comparison, however, the addition of HClO_4_ to an air-saturated PhCN solution of CpFe^II^Trip resulted in a spectral change, as shown in Fig. S13 (in the ESI†). The characteristic absorption bands of CpFe^II^Trip at 545 nm and 608 nm disappeared upon the addition of HClO_4_ with the appearance of new absorption bands at 565 nm, which can be attributed to those of H_2_Trip^+^. This indicates that CpFe^II^Trip was easily demetallated and protonated to afford H_2_Trip^+^ in the presence of HClO_4_, as shown in Fig. S13 (in the ESI†).

### Kinetics and mechanism of the catalytic two-electron reduction of O_2_ by Me_8_Fc with HTrip

**Scheme 3:**
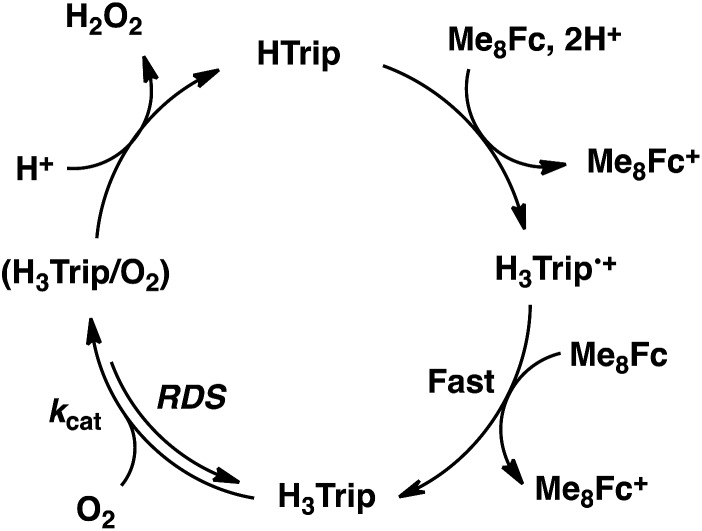


**Fig. 8:**
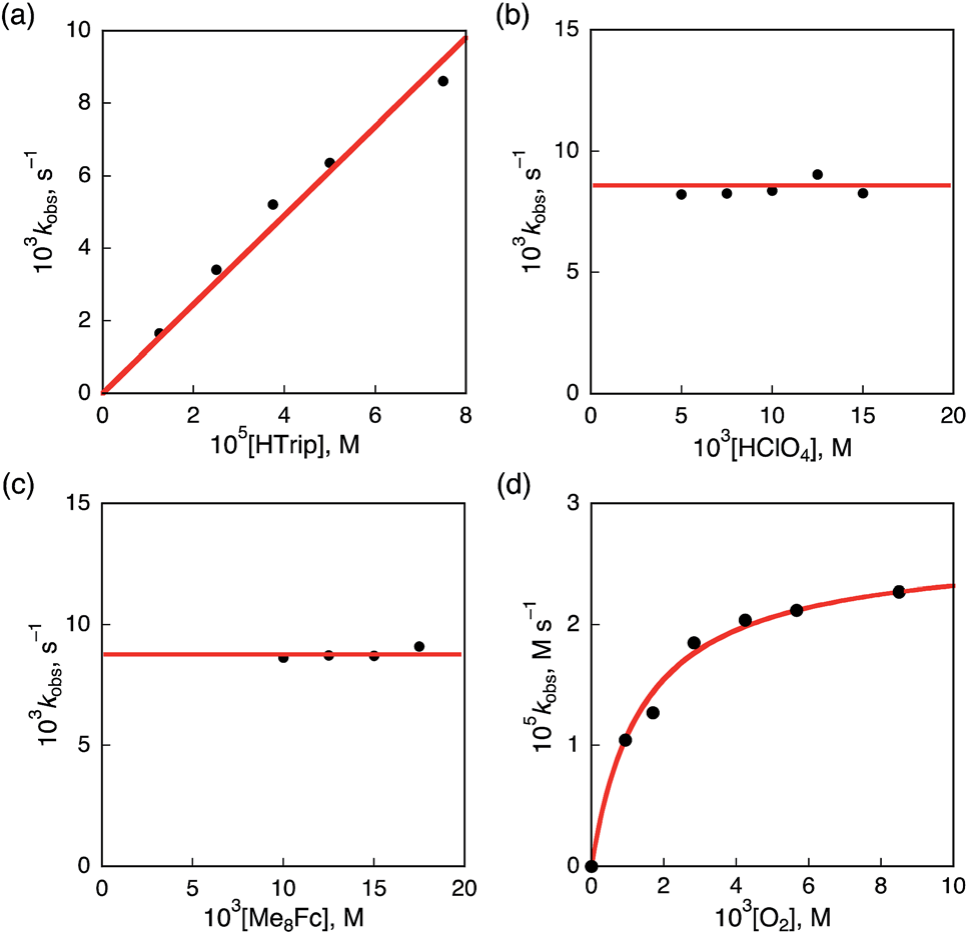
Plots of (a) *k*_obs_*vs.* [HTrip] for the two-electron reduction of O_2_ (9.4 × 10^–4^ M) by Me_2_Fc (1.0 × 10^–2^ M) with various concentrations of HTrip in the presence of HClO_4_ (1.0 × 10^–2^ M) in PhCN; (b) *k*_obs_*vs.* [HClO_4_] for the two-electron reduction of O_2_ (9.4 × 10^–4^ M) by Me_8_Fc (1.0 × 10^–2^ M) with HTrip (5.0 × 10^–5^ M) in PhCN at 298 K; (c) *k*_obs_*vs.* [Me_8_Fc] for the two-electron reduction of O_2_ (9.4 × 10^–4^ M) by various concentrations of Me_8_Fc with HTrip (5.0 × 10^–5^ M) in the presence of HClO_4_ (1.0 × 10^–2^ M) in PhCN at 298 K; and (d) *k*_obs_*vs.* [O_2_] for the two-electron reduction of O_2_ by Me_8_Fc (1.0 × 10^–2^ M) with HTrip (5.0 × 10^–5^ M) in the presence of HClO_4_ (1.0 × 10^–2^ M) in PhCN at 298 K.

The dependence of the first-order rate constant for the formation of Me_8_Fc^+^ on the concentrations of HTrip, HClO_4_, Me_8_Fc, and O_2_ was examined, as shown in Fig. S14 (in the ESI†), where the first-order rate constants were determined from the initial slopes of the first-order plots in order to avoid further complication due to the deactivation of the catalyst during the reactions, as shown in Fig. S15 (in the ESI†). The observed first-order rate constant (*k*_obs_) was proportional to the concentration of HTrip, whereas the *k*_obs_ value remained constant irrespective of the concentration of HClO_4_ or Me_8_Fc (Fig. 8). Although no degradation of HTrip occurred under the present acidic conditions (Fig. S16 in the ESI†), the turnover number (TON) based on HTrip was determined to be more than 40 when the lower concentration of HTrip (1.3 × 10^–5^ M) was employed, as shown in Fig. S14a (in the ESI†). Because the catalytic rate depends only on the concentrations of HTrip and O_2_, the rate-determining step in the catalytic cycle must be the reaction of H_3_Trip with O_2_ in Scheme 3. The dependence of the initial rate of formation of Me_8_Fc^+^ on the concentration of O_2_ shows saturation behaviour at large concentrations of O_2_ (Fig. 8d). Such saturation behaviour is consistent with the formation of the O_2_ complex (H_3_Trip/O_2_) in the oxidation of H_3_Trip with O_2_ (Fig. 6b and Scheme 3). The overall catalytic cycle is shown in Scheme 3, where proton-coupled electron transfer from Me_8_Fc to HTrip is followed by a second electron transfer from Me_8_Fc to H_3_Trip˙^+^ to produce H_3_Trip, which is slowly oxidized by O_2_*via* the H_3_Trip/O_2_ complex as the rate-determining step. Because the direct reaction between H_3_Trip and O_2_ in the H_3_Trip/O_2_ complex is spin-forbidden, the reaction may proceed *via* hydrogen atom transfer from H_3_Trip to O_2_ in the H_3_Trip/O_2_ complex to produce the (H_2_Trip˙/HO_2_˙) intermediate, followed by a rapid second hydrogen transfer from H_2_Trip˙ to HO_2_˙ to yield H_2_O_2_, accompanied by regeneration of HTrip (Scheme 3). According to Scheme 3, the rate of formation of Me_8_Fc^+^ is given by eqn (4),

4 d[Me_8_Fc^+^]/d*t* = *k*_cat_[H_3_Trip/O_2_],

where *k*_cat_ is the rate constant of the hydrogen atom transfer from H_3_Trip to O_2_ in the H_3_Trip/O_2_ complex. Because the concentration of the H_3_Trip/O_2_ complex is given by eqn (5)

5 [H_3_Trip/O_2_] = *K*[HTrip][O_2_]/(1 + *K*[O_2_]),

using the formation constant (*K*), the initial concentration of HTrip, which is converted to H_3_Trip in the catalytic reaction, and the concentration of O_2_, eqn (4) is rewritten as eqn (6).

6 d[Me_8_Fc^+^]/d*t* = *k*_cat_*K*[HTrip][O_2_]/(1 + *K*[O_2_])

This kinetic equation agrees with the experimental observations in Fig. 8. The *k*_cat_ and *K* values were determined from the dependence of the catalytic rate on the concentration of O_2_ (Fig. 8d) to be 0.5 s^–1^ and 8.4 × 10^2^ M^–1^, respectively.

**Fig. 9:**
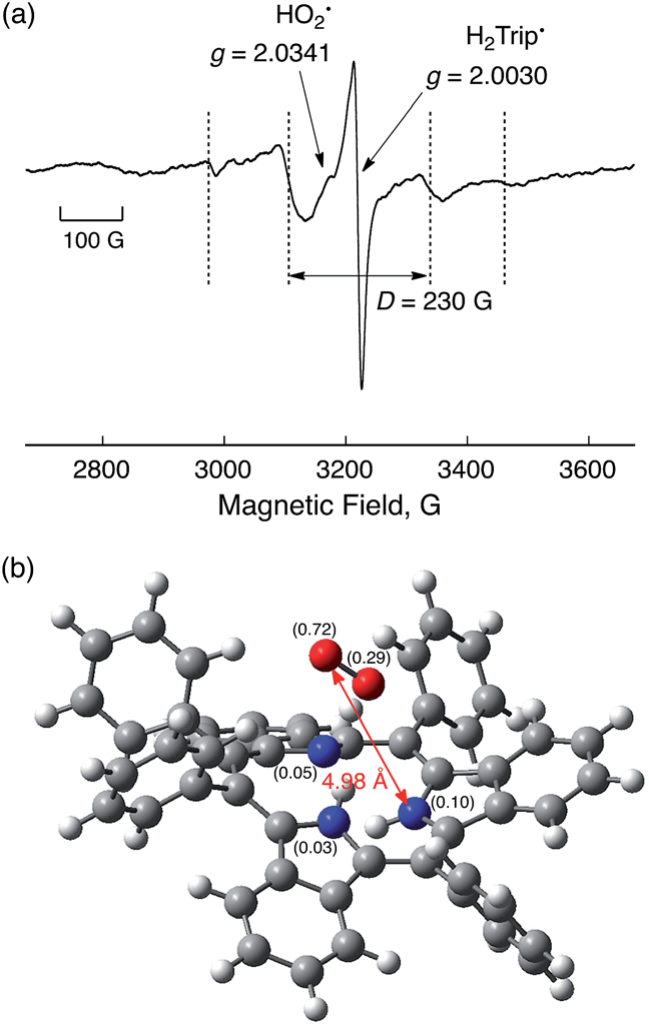
EPR spectrum observed after the reduction of HTrip (1.0 × 10^–3^ M) by (BNA)_2_ (2.0 × 10^–3^ M) in the presence of HClO_4_ (1.0 × 10^–3^ M) in aerated PhCN under photoirradiation using a high-pressure Hg lamp (1000 W) measured at 80 K. Experimental conditions: Microwave frequency 9.0 GHz, microwave power 1.0 mW, modulation frequency 100 kHz, and modulation width 10 G. (b) Optimized structure of H_3_Trip/O_2_ calculated by DFT with calculated spin-density values given in parentheses at the UB3LYP/6-31G(d) level of theory.

Although the radical pair (H_2_Trip˙/HO_2_˙) in Scheme 3 cannot be detected during the catalytic reaction, the formation of the radical pair (H_2_Trip˙/HO_2_˙) was successfully detected by EPR measurements using 1-benzyl-1,4-dihydronicotinamide dimer [(BNA)_2_]^44^ as an electron donor to produce H_3_Trip under photoirradiation at low temperature. The observed EPR spectrum in aerated PhCN in the presence of HClO_4_ at low temperature is shown in Fig. 9. A triplet fine structure EPR signal was observed as well as the typical anisotropic signals for HO_2_˙ with the *g*_||_ value of 2.0341, and isotropic signals for H_2_Trip˙ at 2.0030.^45^,^46^ From the zero-field splitting value (*D* = 230 G), the distance (*r*) between two unpaired electrons was determined using the relation *D* = 27 800/*r*^3^ 47 to be 4.9 Å. This distance is consistent with the estimated distance between O_2_ and H_3_Trip in the H_3_Trip/O_2_ complex by DFT calculations (Fig. 9b).

## Conclusion

Metal-free triphyrin acts as an efficient catalyst for the two-electron reduction of O_2_ by Me_8_Fc to produce H_2_O_2_ in the presence of HClO_4_ in PhCN at 298 K. The rate-determining step (RDS) in the catalytic cycle has been found to be hydrogen atom transfer from H_3_Tip to O_2_ in the H_3_Trip/O_2_ complex to produce the radical pair (H_3_Trip˙^+^/HO_2_˙), which was detected as a triplet species by EPR at 80 K. The distance between the two unpaired electrons (4.9 Å) determined from the zero-field splitting constant (*D*) agrees with the distance in the H_3_Trip/O_2_ complex calculated by DFT. The present study provides valuable insight into the catalytic mechanism of the two-electron reduction of O_2_ with an organic catalyst, and may lead to the development of more efficient metal-free organic catalysts for the selective two-electron reduction of O_2_ to produce H_2_O_2_.

## Experimental section

### General procedure

Chemicals were purchased from commercial sources and used without further purification, unless otherwise noted. Perchloric acid (HClO_4_, 70%), trifluoroacetic acid (TFA), ferrocene (Fc), and 1,1-dimethylferrocene (Me_2_Fc) were purchased from Wako Pure Chemical Industries Ltd. Octamethylferrocene (Me_8_Fc) and decamethylferrocene (Me_10_Fc) were received from Sigma Aldrich. Fc, Me_2_Fc, Me_8_Fc, and Me_10_Fc were purified by sublimation or recrystallization from ethanol. Benzonitrile (PhCN) used for spectroscopic and electrochemical measurements was distilled over phosphorus pentoxide prior to use.^48^ [14]Triphyrin(2.1.1) [HTrip] was synthesized according to the reported procedure.^41^ Fe(ii)(TMC)(OTf)_2_ (TMC = 1,4,8,11-tetramethyl-1,4,8,11-tetraazacyclotetradecane; OTf = CF_3_SO_3_) was prepared according to a literature method.^43^ Tetra-*n*-butylammonium hexafluorophosphate (TBAPF_6_) was twice recrystallized from ethanol and dried *in vacuo* prior to use. ^1^H NMR spectra (300 MHz) were recorded on a JEOL AL-300 spectrometer at room temperature and chemical shifts (ppm) were determined relative to tetramethylsilane (TMS). UV-vis absorption spectroscopy was carried out on a Hewlett Packard 8453 diode array spectrophotometer at room temperature using a quartz cell (light path length = 1 cm).

### Spectroscopic measurements

The amount of hydrogen peroxide (H_2_O_2_) produced was determined by titration with iodide ion: a dilute CH_3_CN solution (2.0 mL) of the product mixture (50 μL) was treated with an excess amount of NaI, and the amount of I_3_^–^ formed was determined from the absorption spectrum (*λ*_max_ = 361 nm, *ε* = 2.8 × 10^4^ M^–1^ cm^–1^).^49^ The formation of H_2_O_2_ in the catalytic O_2_ reduction with HTrip was again confirmed by the reaction between H_2_O_2_ and Fe(ii)(TMC)(OTf)_2_ to afford the corresponding Fe(iv)-oxo species. The amount of the Fe(iv)-oxo species produced was determined from the absorption spectrum (*λ*_max_ = 820 nm, *ε* = 400 M^–1^ cm^–1^).^43^

The turnover numbers (TON = the number of moles of H_2_O_2_ formed per mole of HTrip in the catalytic two-electron reduction of O_2_) were determined from the concentration of produced Me_8_Fc^+^ under catalytic conditions, where stoichiometric production of H_2_O_2_ was confirmed by iodometric titration.

### Kinetic measurements

Rate constants of oxidation of ferrocene derivatives by O_2_ in the presence of a catalytic amount of HTrip and an excess amount of HClO_4_ in PhCN at 298 K were determined by monitoring the appearance of an absorption band due to the corresponding ferrocenium ions (Fc^+^, *λ*_max_ = 620 nm, *ε*_max_ = 330 M^–1^ cm^–1^; Me_2_Fc^+^, *λ*_max_ = 650 nm, *ε*_max_ = 290 M^–1^ cm^–1^; Me_8_Fc^+^, *λ*_max_ = 750 nm, *ε*_max_ = 410 M^–1^ cm^–1^; Me_10_Fc^+^, *λ*_max_ = 780 nm, *ε*_max_ = 450 M^–1^ cm^–1^).^14^ At the wavelengths monitored, spectral overlap was observed with H_3_Trip (*λ* = 738 nm (*ε* = 1.6 × 10^3^ M^–1^ cm^–1^)), H_3_Trip/O_2_ (*λ* = 720 nm (*ε* = 1.2 × 10^3^ M^–1^ cm^–1^)). The concentration of O_2_ in an air-saturated PhCN solution was determined to be 1.7 × 10^–3^ M as reported previously.^50^ The concentrations of ferrocene derivatives employed for the catalytic reduction of O_2_ were much larger than that of O_2_, as O_2_ is the rate-limiting reagent in the reaction solution. The PhCN solutions containing various concentrations of O_2_ for the kinetic measurements were prepared by N_2_/O_2_ mixed gas bubbling using a KOFLOC GASBLENDER GB-3C. Typically, a PhCN stock solution of a ferrocene derivative was added using a microsyringe to a PhCN solution containing HTrip and HClO_4_ in a quartz cuvette (light path length = 1 cm).

### Electrochemical measurements

Cyclic voltammetry (CV) measurements were performed on an ALS 630B electrochemical analyser and voltammograms were measured in deaerated PhCN containing 0.10 M TBAPF_6_ as a supporting electrolyte at room temperature. A conventional three-electrode cell was used with a glassy carbon working electrode (surface area of 0.3 mm^2^) and a platinum wire as the counter electrode. The glassy carbon working electrode (BAS) was routinely polished with BAS polishing alumina suspension and rinsed with acetone before use. The potentials were measured with respect to the Ag/AgNO_3_ (1.0 × 10^–2^ M) reference electrode. All potentials (*vs.* Ag/AgNO_3_) were converted to values *vs.* SCE by adding 0.29 V.^51^ Redox potentials were determined using the relation *E*_1/2_ = (*E*_pa_ + *E*_pc_)/2.

### Spectroelectrochemical measurements

UV-visible spectroelectrochemical experiments were performed with a home-built thin-layer cell (1 mm) that had a light transparent platinum net working electrode. Potentials were applied and monitored with an ALS 730D electrochemical analyser.

### EPR measurements

EPR spectra were measured on a JEOL X-band EPR spectrometer (JES-ME-LX) using a quartz EPR tube containing a deaerated frozen sample solution at 80 K. The internal diameter of the EPR tube is 4.0 mm, which is small enough to fill the EPR cavity but large enough to obtain good signal-to-noise ratios during the EPR measurements at low temperatures (at 80 K). EPR spectrum of HTrip˙^–^ produced by the electrochemical reduction of HTrip was measured using a home-built three-electrode quartz EPR tube. Potentials were applied and monitored with an ALS 730D electrochemical analyser. EPR spectra were measured under nonsaturating microwave power conditions. The amplitude of modulation was chosen to optimize the resolution and the signal-to-noise (*S*/*N*) ratio of the observed spectra. The *g* values were calibrated with a Mn^2+^ marker.

### Theoretical calculations

Density functional theory (DFT) calculations were performed on a 32CPU workstation (PQS, Quantum Cube QS8-2400C-064). Geometry optimisations were carried out using the B3LYP/6-31G(d) level of theory^52^ for HTrip˙^–^, H_2_Trip^+^, H_3_Trip^2+^, H_3_Trip˙^+^, and [H_3_Trip/O_2_]. All calculations were performed using Gaussian 09, revision A.02.^53^ Graphical outputs of the computational results were generated with the *GaussView* software program (ver. 3.09) developed by Semichem, Inc.^54^

## Supplementary Material

Supplementary information
