# Supplementary material for: Catalytic two-electron reduction of dioxygen catalysed by metal-free [14]triphyrin(2.1.1)
Source: Chem Sci. 2015 Aug 3;6(11):6496–504. doi: 10.1039/c5sc02465j (PMC6054055; doi:10.1039/c5sc02465j)
Supplement: Supplementary file 1 [file SC-006-C5SC02465J-s001.pdf]

Electronic Supplementary Material (ESI) for Chemical Science.
This journal is © The Royal Society of Chemistry 2015

Electronic supplementary information (ESI)

Catalytic two-electron reduction of dioxygen catalysed by metal-free [14]triphyrin(2.1.1)

Kentaro Mase,[a] Kei Ohkubo,[a,b] Xue Zhaoli,[c] Hiroko Yamada*,[c] and Shunichi Fukuzumi*,[a,b,d]

[a]*Department of Material and Life Science, Graduate School of Engineering, ALCA and SENTAN, Japan Science and Technology Agency (JST), Osaka University, Suita, Osaka 565-0871, Japan*

[b]*Department of Chemistry and Nano Science, Ewha Womans University, Seoul 120-750, Korea*

[c]*Graduate School of Materials Science, Nara Institute of Science and Technology, CREST, Japan Science and Technology Agency (JST), Ikoma, Nara 630-0192, Japan*

[d]*Faculty of Science and Engineering, ALCA, SENTAN, Japan Science and Technology Agency (JST), Meijo University, Nagoya, Aichi 468-0073, Japan*

Corresponding Author

fukuzumi@chem.eng.osaka-u.ac.jp

hyamada@ms.naist.jp

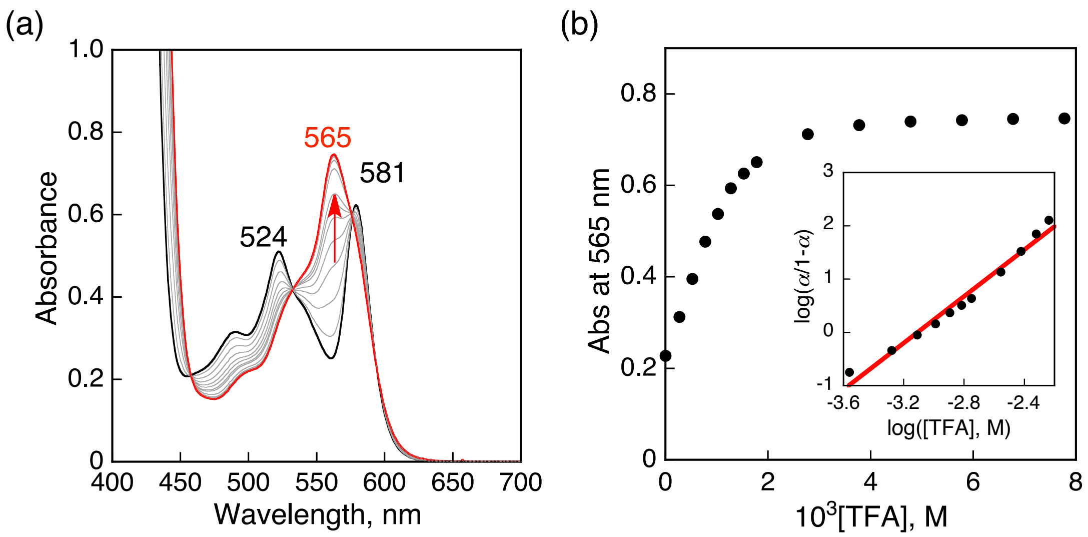

Fig. S1 (a) Absorption spectral changes of HTrip (3.0×10^{-5} M) upon the addition of TFA in air-saturated PhCN at 298 K. (b) Absorbance change profile at 565 nm. Inset shows Hill plot of absorbance change at 565 nm upon addition of TFA. $\alpha = (A - A_0)/(A_\infty - A_0)$.

Comment. The pK_a value of H_2Trip^+ in PhCN was determined from the titration of HTrip with trifluoroacetic acid (TFA) as given by eq 1, where K is protonation equilibrium constant of HTrip. The K value for the monoprotonation of HTrip was determined to be 1.3×10^3 M^{-1} in

$$\mathrm{HTrip} + \mathrm{CF_3COOH} \overset{K}{\rightleftarrows} \mathrm{HTrip} + \mathrm{CF_3COOH} \qquad (1)$$

air-saturated PhCN at 298K, as shown in Fig. S1b. In order to determine the pK_a value of H_2Trip^+, K is rewritten by eq2, where K_a and K_a' are dissociation constants of H_2Trip^+ and

$$K = K_a/K_a' \qquad (2)$$

CF_3COOH, respectively. The pK_a' value was previously reported to be 12.65 in MeCN at 298 K.[1] Thus, the pK_a value was estimated to be 15.8 according to eq3.

$$\mathrm{p}K_a = \mathrm{p}K_a' - \mathrm{p}K \qquad (3)$$

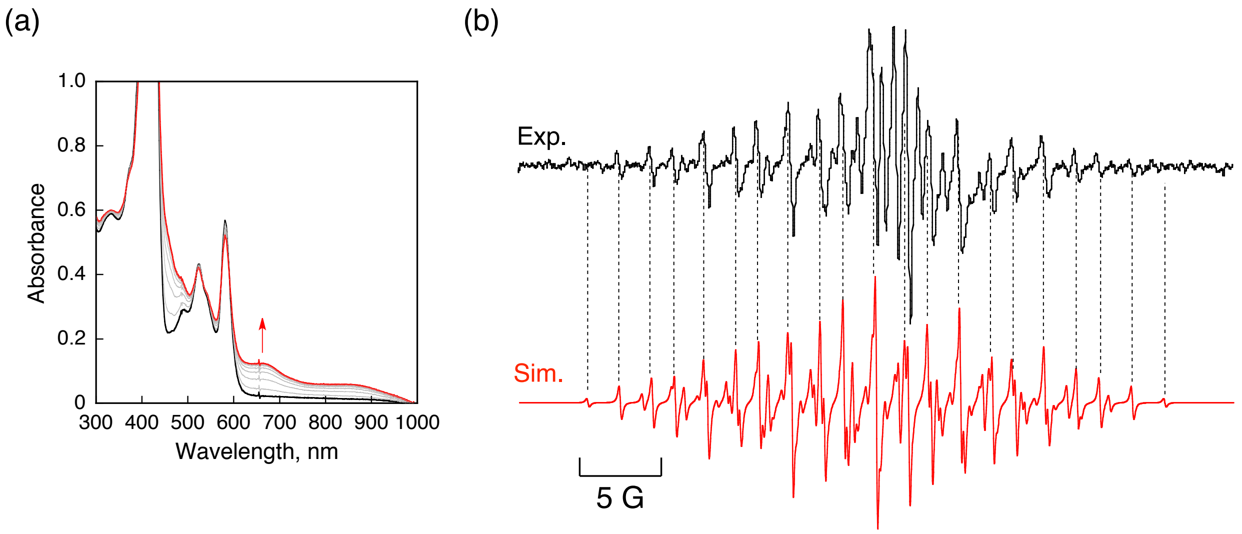

Fig. S2 (a) Thin-layer UV-visible spectral changes of HTrip (2.5×10^{-4} M) in deaerated PhCN containing 0.1 M $TBAPF_6$ in the electrochemical one-electron reduction of HTrip at a controlled potential of –1.25 V vs SCE at 298 K. (b) EPR spectrum of $HTrip^{\bullet-}$ produced by the electrochemical reduction of HTrip (2.5×10^{-4} M) in deaerated PhCN at a controlled potential of –1.25 V vs SCE at 298 K. The black and red lines show experimental and simulated spectra using *hfc* values (a(2N) = 5.31 G, a(2H) = 3.48 G, a(2H) = 2.05 G, a(2H) = 2.04 G), respectively. Experimental parameters: microwave frequency 9.4 GHz, microwave power 1.0 mW, modulation frequency 100 kHz, and modulation width 0.35 G.

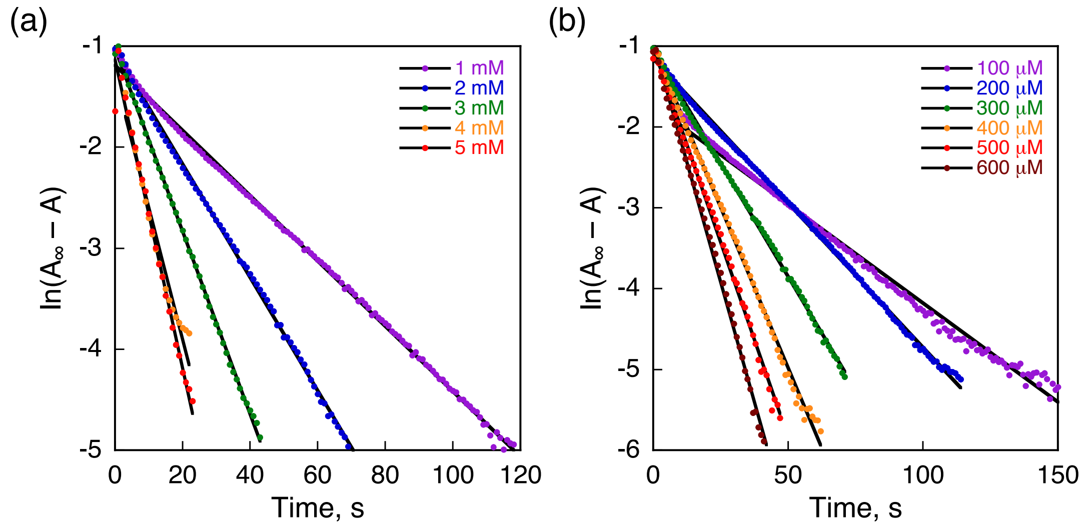

Fig. S3 Pseudo-first-order plots for the reduction of H_2Trip^+ (2.5×10^{-5} M) by (a) various concentration of Me_8Fc in the presence of $HClO_4$ (3.0×10^{-4} M) and (b) by Me_8Fc (2.0×10^{-3} M) in the presence of various concentration of $HClO_4$ in deaerated PhCN at 298 K. A and A_∞ represent absorbance at 738 nm during the reactions and final absorbance at 738 nm, respectively.

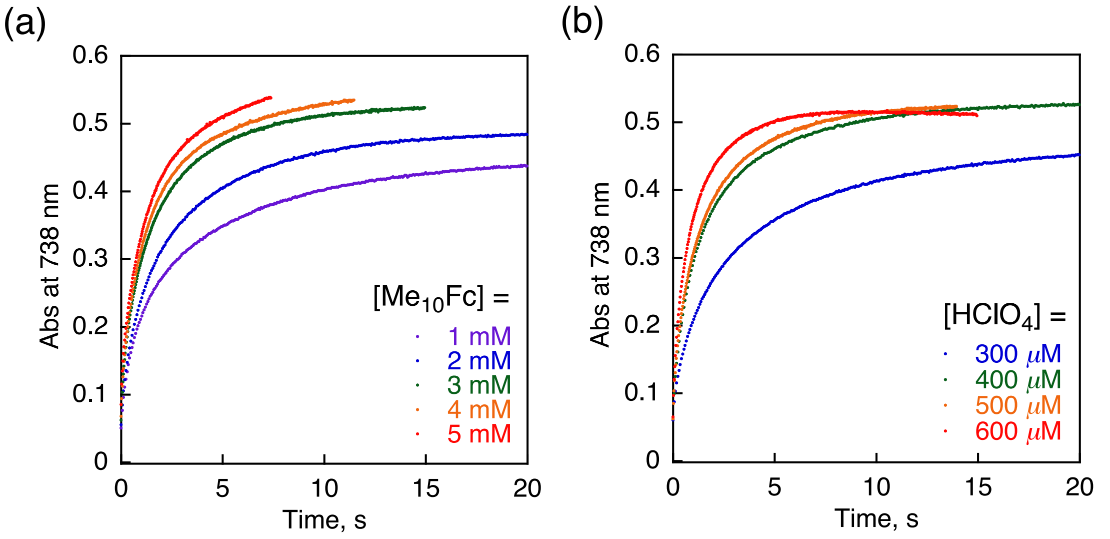

Fig. S4 Time profiles of absorbance at 738 nm due to H_3Trip in the reduction of H_2Trip$^+$ (2.5×10^{-5} M) (a) by various concentrations of Me_{10}Fc in the presence of $HClO_4$ (5.0×10^{-4} M) and (b) by Me_{10}Fc (3.0×10^{-3} M) in the presence of various concentrations of $HClO_4$ in deaerated PhCN at 298 K.

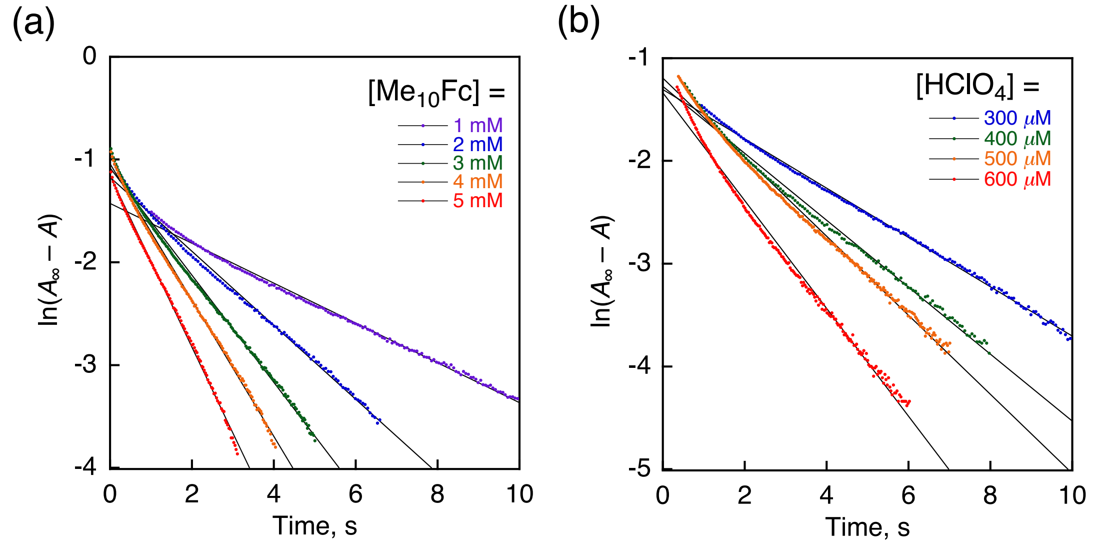

Fig. S5 Pseudo-first-order plots for the reduction of H_2Trip$^+$ (2.5×10^{-5} M) by (a) various concentration of Me_{10}Fc in the presence of $HClO_4$ (5.0×10^{-4} M) and (b) by Me_{10}Fc (3.0×10^{-3} M) in the presence of various concentration of $HClO_4$ in deaerated PhCN at 298 K. A and A_∞ represent absorbance at 738 nm during the reactions and final absorbance at 738 nm, respectively.

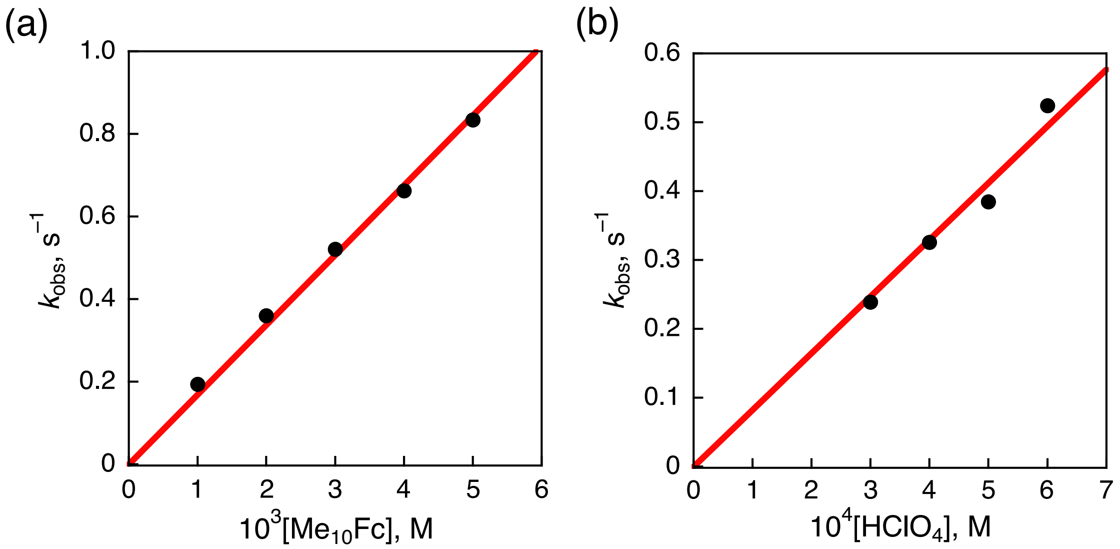

Fig. S6 Plot of (a) k_{obs} vs [$Me_{10}Fc$] for the reduction of H_2Trip^+ (2.5×10^{-5} M) by various concentrations of $Me_{10}Fc$ in the presence of $HClO_4$ (5.0×10^{-4} M) in PhCN at 298 K. (b) Plot of k_{obs} vs [$HClO_4$] for the reduction of H_2Trip^+ (2.5×10^{-5} M) by $Me_{10}Fc$ (3.0×10^{-3} M) in the presence of various concentrations of $HClO_4$ in deaerated PhCN at 298 K.

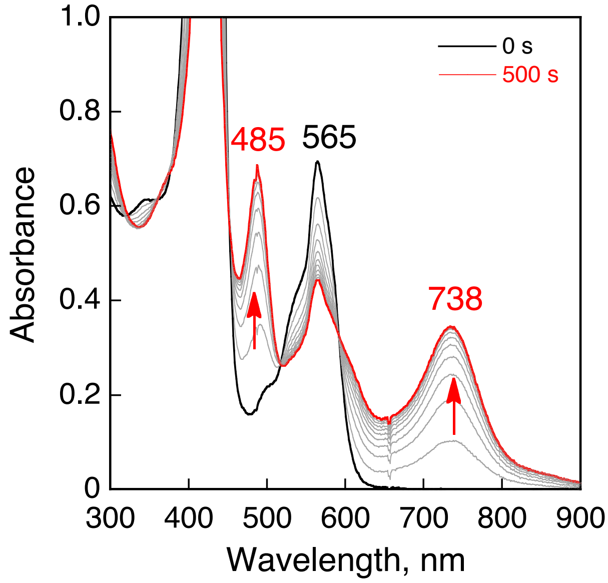

Fig. S7 Thin-layer UV-visible spectral changes of H_2Trip^+ (2.5×10^{-4} M) in the presence of $HClO_4$ (1.0×10^{-4} M) in PhCN containing 0.1 M $TBAPF_6$ in the electrochemical reduction of H_2Trip^+ at a controlled potential of –0.30 V vs SCE.

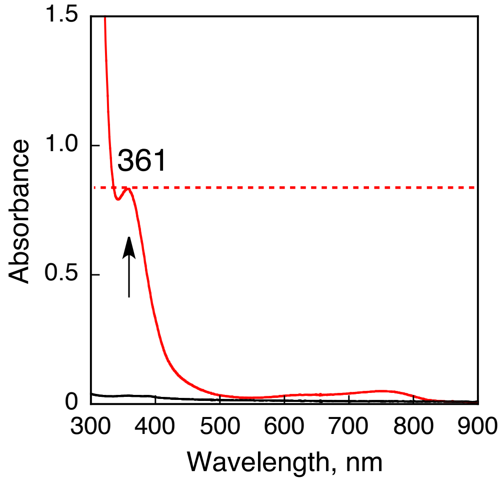

Fig. S8 Absorption spectral change before (black) and after (red) the addition of NaI (excess) to a diluted reaction mixture in MeCN containing HTrip (1.3×10^{-6} M), Me_8Fc^+ (4.7×10^{-5} M) and $HClO_4$ (2.5×10^{-4} M). The dotted line is the absorbance at 361 nm due to I_3^- of 2.4×10^{-5} M.

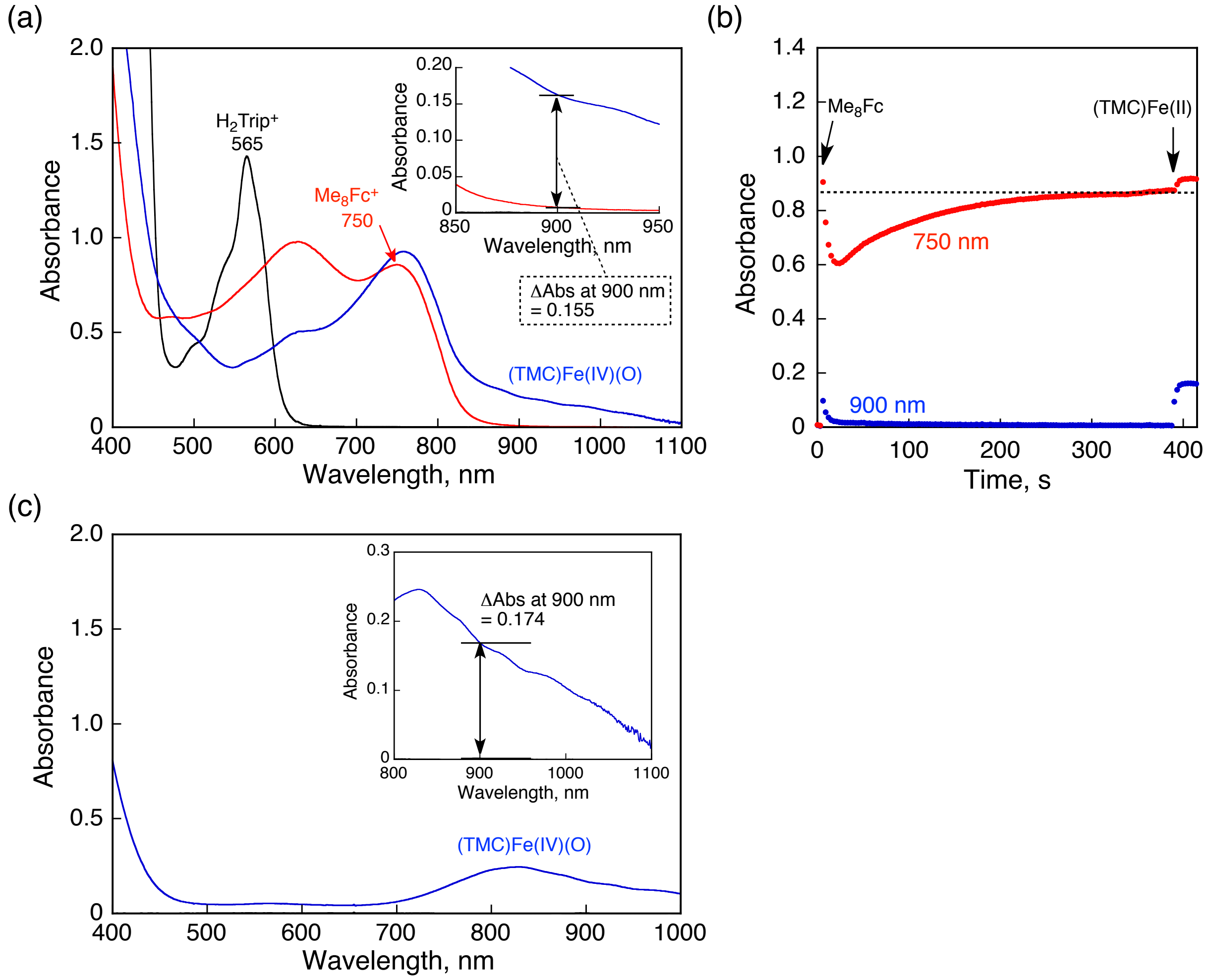

Fig. S9 Absorption spectral change in the two-electron reduction of O_2 (8.5×10^{-3} M) by Me_8Fc (2.0×10^{-3} M) with HTrip (5.0×10^{-5} M) in the presence of $HClO_4$ (1.0×10^{-2} M) in O_2-saturated PhCN at 298 K. The black and red lines show the spectra before and after addition of Me_8Fc, respectively. The blue line shows the spectrum after addition of $[(TMC)Fe^{II}](OTf)_2$ (TMC = 1,4,8,11-tetramethyl-1,4,8,11-tetraazacyclotetradecane: 1.3×10^{-3} M) to the reaction solution (red line). (b) Time profile of absorbance at 750 nm and 900 nm due to the formation of Me_8Fc^+ and $[(TMC)Fe^{IV}(O)]^{2+}$, respectively. The dotted line is the absorbance at 750 nm due to 2.0×10^{-3} M of Me_8Fc^+. Me_8Fc (2.0×10^{-3} M) and $[(TMC)Fe^{II}](OTf)_2$ (1.3×10^{-3} M) were added at t = 0 and 390 s, respectively. (c) Absorption spectral change in the reaction of $[(TMC)Fe^{II}](OTf)_2$ (1.3×10^{-3} M) with H_2O_2 (1.0×10^{-3} M) in the presence of $HClO_4$ (1.0×10^{-2} M) in N_2-saturated PhCN at 298 K.

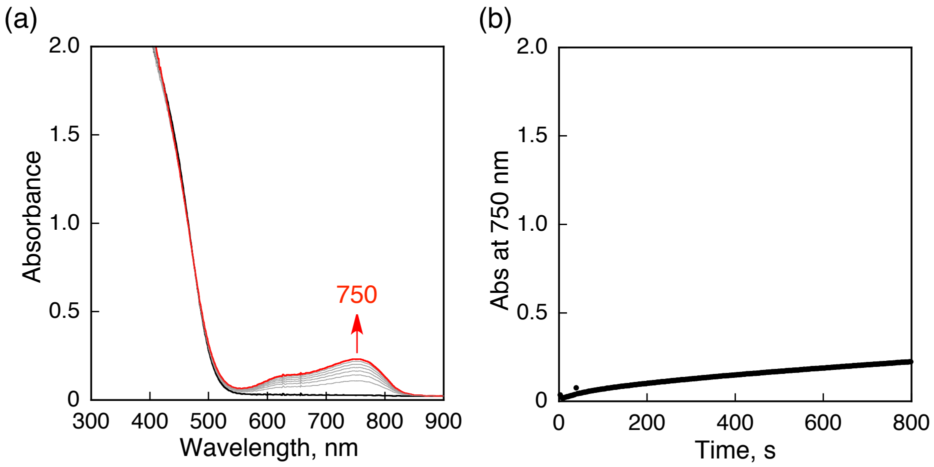

Fig. S10 (a) Absorption spectral changes in the reduction of O_2 (9.4×10^{-4} M) by Me_8Fc (1.0×10^{-2} M) in the presence of $HClO_4$ (1.0×10^{-2} M) in PhCN at 298 K. The black and red lines show the spectra before and after addition of $HClO_4$, respectively. (b) Time profile of absorbance at 750 nm due to the formation of Me_8Fc^+.

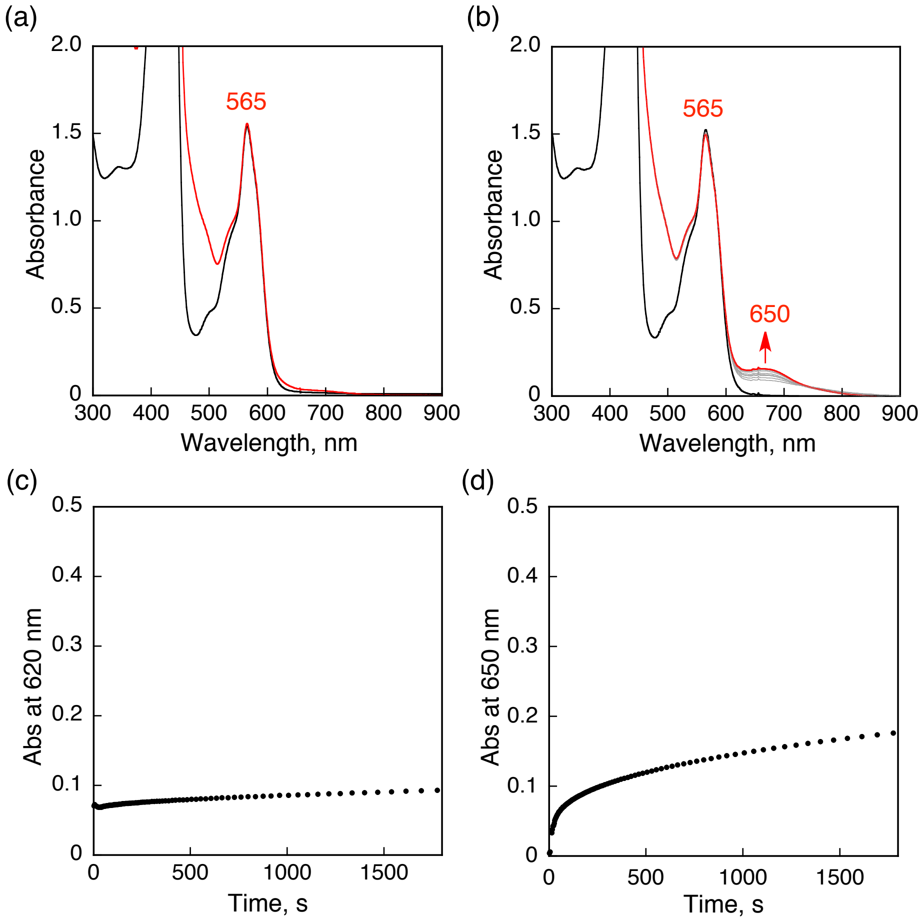

Fig. S11 Absorption spectral changes in the two-electron reduction of O_2 (9.4×10^{-4} M) by (a) Fc (1.0×10^{-2} M) and (b) Me_2Fc (1.0×10^{-2} M) with HTrip (5.0×10^{-5} M) in the presence of $HClO_4$ (1.0×10^{-2} M) in PhCN at 298 K. The black and red lines show the spectra before and after addition of ferrocene derivatives, respectively. Time profiles of (c) absorbance at 620 nm due to Fc^+ and (d) absorbance at 650 nm due to Me_2Fc^+.

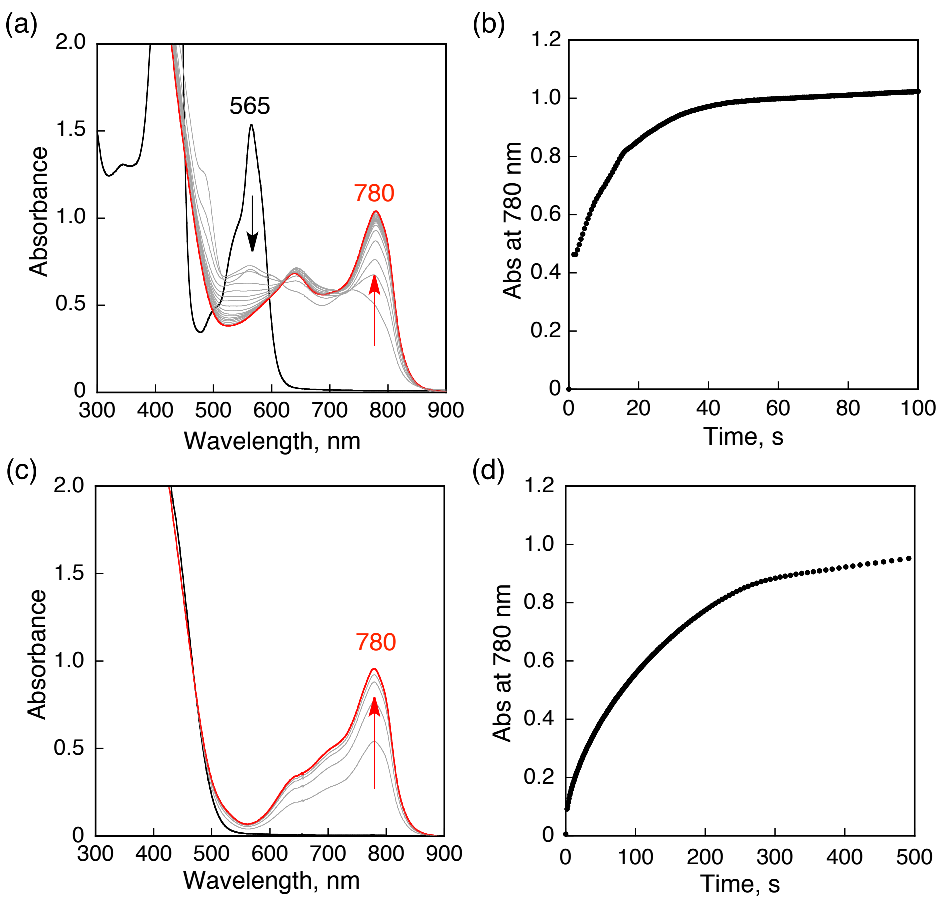

Fig. S12 (a) Absorption spectral changes and (b) time profiles of absorbance at 780 nm due to $Me_{10}Fc^+$ in the two-electron reduction of O_2 (9.4 × 10^{-4} M) by $Me_{10}Fc$ (1.0 × 10^{-2} M) with HTrip (5.0 × 10^{-5} M) in the presence of $HClO_4$ (1.0 × 10^{-2} M) in PhCN at 298 K. (c) Absorption spectral changes and (d) time profiles of absorbance at 780 nm due to $Me_{10}Fc^+$ in the two-electron reduction of O_2 (9.4 × 10^{-4} M) by $Me_{10}Fc$ (1.0 × 10^{-2} M) without HTrip in the presence of $HClO_4$ (1.0 × 10^{-2} M) in PhCN at 298 K. The black and red lines show the spectra before and after addition of ferrocene derivatives, respectively.

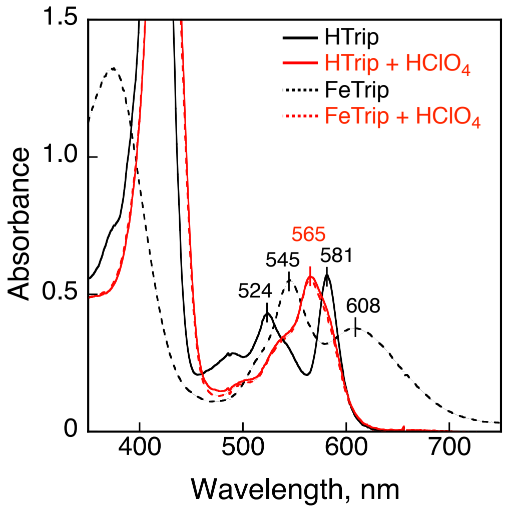

Fig. S13 Absorption spectral changes of HTrip (2.0 × 10^{-5} M) (solid line) and FeTrip (2.0 × 10^{-5} M) (dashed line) in air-saturated PhCN at 298 K. The black and red lines show the spectra before and after addition of $HClO_4$ (1.0 × 10^{-2} M), respectively.

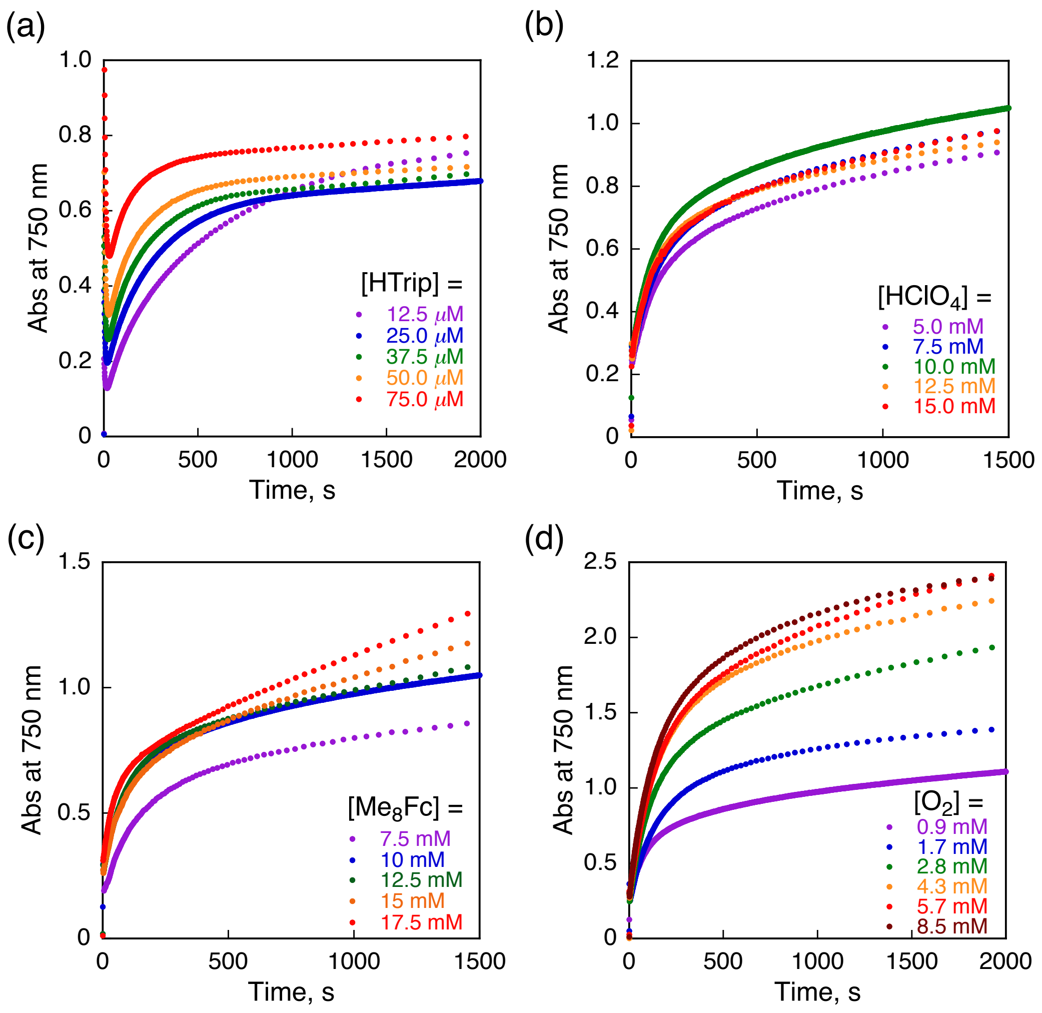

Fig. S14 Time profiles of absorbance at 750 nm due to Me_8Fc^+ in the two-electron reduction of (a) O_2 (9.4×10^{-4} M) by Me_8Fc (1.0×10^{-2} M) with various concentrations of HTrip in the presence of $HClO_4$ (1.0×10^{-2} M) in PhCN at 298 K. (b) Those of O_2 (9.4×10^{-4} M) by Me_8Fc (1.0×10^{-2} M) with HTrip (5.0×10^{-5} M) in the presence of various concentrations of $HClO_4$ in PhCN at 298 K. (c) Those of O_2 (9.4×10^{-4} M) by various concentrations of Me_8Fc with HTrip (5.0×10^{-5} M) in the presence of $HClO_4$ (1.0×10^{-2} M) in PhCN at 298 K. (d) Those of various concentrations of O_2 (9.4×10^{-4} M) by Me_8Fc (1.0×10^{-2} M) with HTrip (5.0×10^{-5} M) in the presence of $HClO_4$ (1.0×10^{-2} M) in PhCN at 298 K.

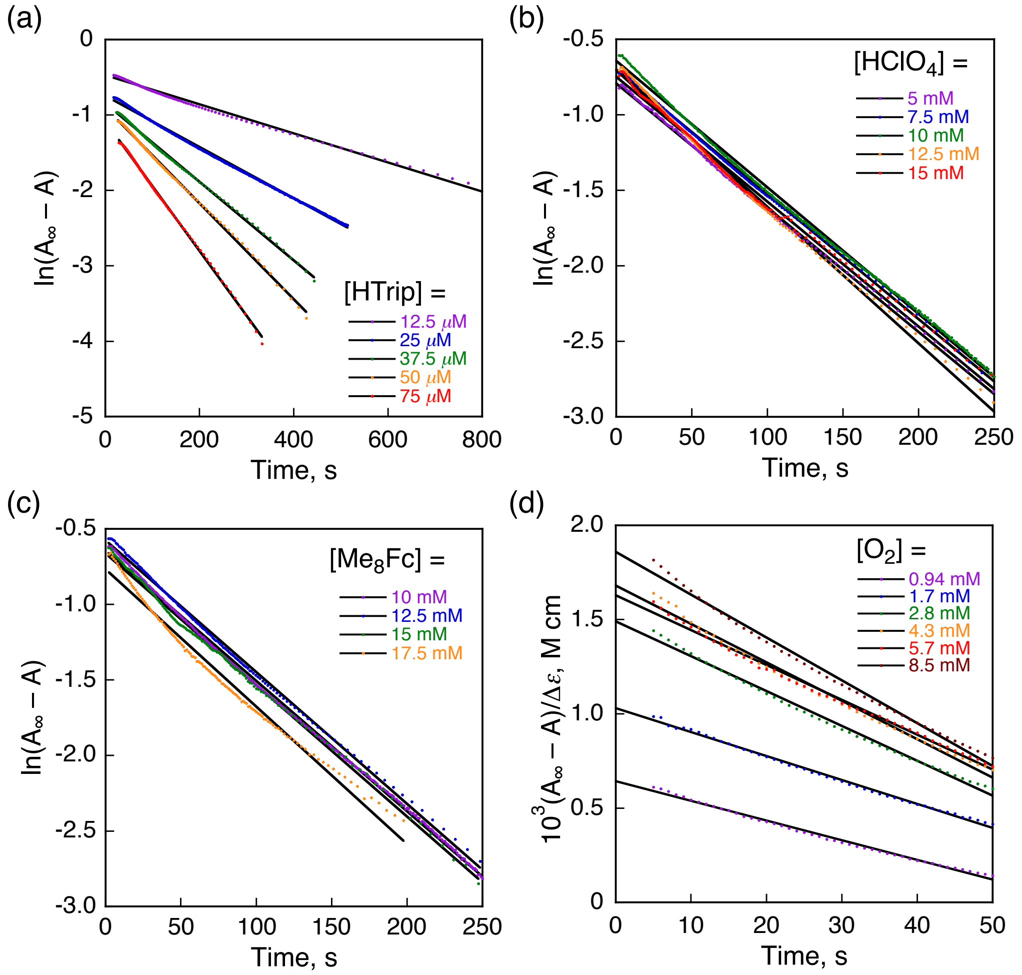

Fig. S15 Pseudo-first-order plots for the two-electron reduction of (a) O_2 (9.4×10^{-4} M) by Me_8Fc (1.0×10^{-2} M) with various concentrations of HTrip in the presence of $HClO_4$ (1.0×10^{-2} M) in PhCN at 298 K. (b) Those of O_2 (9.4×10^{-4} M) by Me_8Fc (1.0×10^{-2} M) with HTrip (5.0×10^{-5} M) in the presence of various concentrations of $HClO_4$ in PhCN at 298 K. (c) Those of O_2 (9.4×10^{-4} M) by various concentrations of Me_8Fc with HTrip (5.0×10^{-5} M) in the presence of $HClO_4$ (1.0×10^{-2} M) in PhCN at 298 K. (d) Those of various concentrations of O_2 (9.4×10^{-4} M) by Me_8Fc (1.0×10^{-2} M) with HTrip (5.0×10^{-5} M) in the presence of $HClO_4$ (1.0×10^{-2} M) in PhCN at 298 K.

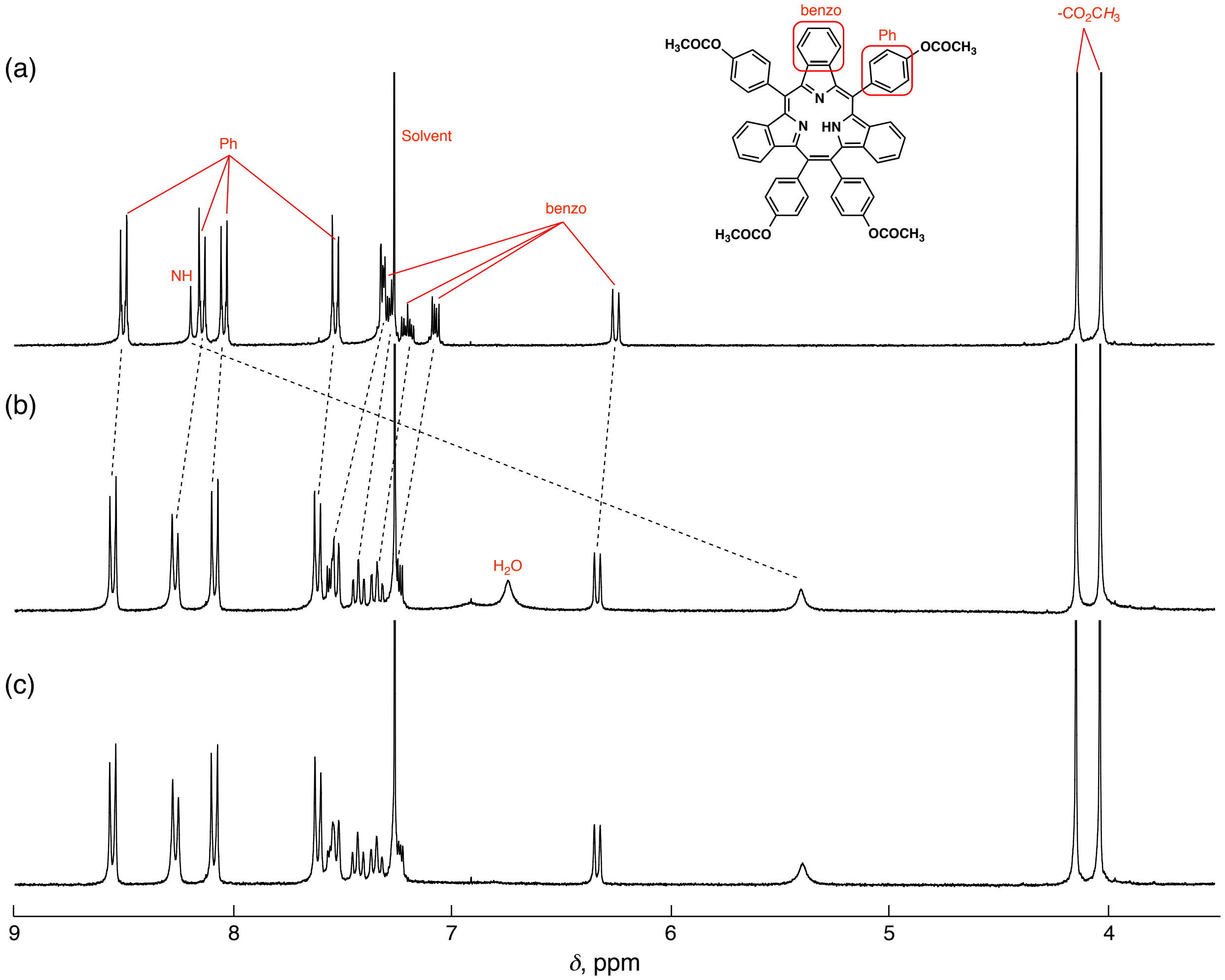

Fig. S16 ^{1}H NMR spectra of (a) HTrip (4.0×10^{-3} M), (b) HTrip (4.0×10^{-3} M) in the presence of $HClO_4$ (1.2×10^{-2} M), and (c) HTrip (4.0×10^{-3} M) in the presence of $HClO_4$ (1.2×10^{-2} M) and Me_8Fc (8.0×10^{-3} M) in air-saturated $CDCl_3$.

Reference

F. Eckert, I. Leito, I. Kaljurand, A. Kütt, A. Klant and M. Diedenhofen, *J. Comp. Chem.*, 2009, **30**, 799-810.

Optimised structure of H_3Trip^{2+}

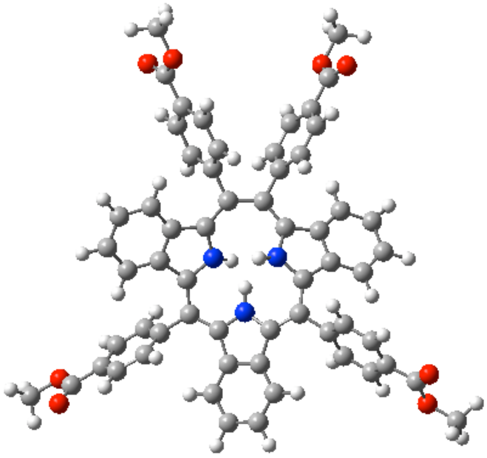

Cartesian coordinates of H_3Trip^{2+} calculated by DFT at the B3LYP/6-31G(d) level of theory

C	3.02053963	1.19481912	0.22795231
C	4.42238909	0.79683944	0.13003934
C	4.45286806	-0.58377495	-0.26107219
C	3.07188908	-1.05550103	-0.31625594
C	2.44585140	-2.31234339	-0.47051420
C	1.03446080	-2.44489867	-0.27285179
C	0.33072896	-3.46972611	0.45534738
C	-0.97368455	-2.96619149	0.76641007
C	-1.11467708	-1.65795818	0.14819547
C	-2.21082727	-0.76346087	0.15879352
C	-2.24796473	0.64542723	-0.17006263
C	-1.19698811	1.59262549	-0.17547880
C	-1.12837423	2.90346024	-0.79997630
C	0.15429925	3.47107605	-0.50983368
C	0.91743501	2.48463526	0.21249232
C	2.33593973	2.42071611	0.38692301
C	5.61655302	1.49325382	0.34849487
H	5.61309611	2.52260477	0.68231339
C	6.82279578	0.83484447	0.12100135
H	7.75637213	1.36330882	0.28630209
C	6.85080206	-0.49263981	-0.33335970
H	7.80536881	-0.96962210	-0.53196293
C	5.67471626	-1.21520852	-0.52040052
H	5.71491855	-2.24317497	-0.85620590
C	0.76216686	-4.71032568	0.96413064
H	1.73808911	-5.10646838	0.71989047
C	-0.09928847	-5.42718138	1.77760354
H	0.20860521	-6.39399398	2.16309471
C	-1.36623155	-4.91453525	2.12223038
H	-2.01172915	-5.48838020	2.77981512
C	-1.80868378	-3.69340101	1.63614763
H	-2.78103685	-3.32097935	1.92461917

C	-2.00994852	3.58490881	-1.66083381
H	-2.96710699	3.16437828	-1.93331480
C	-1.63384203	4.82298928	-2.15957865
H	-2.31527120	5.36105592	-2.81112714
C	-0.38823608	5.39786967	-1.83605792
H	-0.13202217	6.37551633	-2.23192652
C	0.51791446	4.72823143	-1.03083763
H	1.47722155	5.17159953	-0.80333008
C	3.07265592	3.70445105	0.52871275
C	2.82424830	4.51864432	1.64686634
H	2.12775513	4.18749276	2.41203022
C	3.47871215	5.74028371	1.79179464
H	3.29632266	6.35989100	2.66231084
C	4.37547240	6.17432542	0.80819742
C	4.61515979	5.37426212	-0.31761903
H	5.30333205	5.73716373	-1.07411523
C	3.97450066	4.14790206	-0.45672048
H	4.15367925	3.54248295	-1.34052393
C	-3.59371581	1.21157797	-0.50256023
C	-4.30409978	0.76415159	-1.62888313
H	-3.88074060	-0.00689978	-2.26564740
C	-5.54136391	1.31706629	-1.94902530
H	-6.08040582	0.98109143	-2.82748101
C	-6.09909001	2.30886725	-1.13103979
C	-5.40232889	2.74840872	0.00174762
H	-5.85523683	3.51196449	0.62550675
C	-4.15637616	2.21353603	0.30753967
H	-3.62020195	2.56485443	1.18478132
C	-3.52210286	-1.39651570	0.50725952
C	-4.04385195	-2.42428561	-0.29795889
H	-3.50238580	-2.74625598	-1.18318475
C	-5.25668848	-3.02257212	0.02274892
H	-5.67843622	-3.80681972	-0.59717278
C	-5.95944770	-2.62195676	1.16623334
C	-5.44200792	-1.60476263	1.97947444
H	-5.98525076	-1.29880060	2.86627538
C	-4.23908413	-0.98846914	1.64436539
H	-3.84693439	-0.19838476	2.27784624
C	3.23609710	-3.55899171	-0.64442651
C	4.19311178	-3.96639079	0.30294060
H	4.37745031	-3.35802698	1.18367487
C	4.88486115	-5.16353935	0.13487620
H	5.61244538	-5.48354394	0.87218194
C	4.63604459	-5.96740214	-0.98630658
C	3.68343151	-5.56756300	-1.93121751
H	3.50723141	-6.20406989	-2.79198256
C	2.98179818	-4.37892485	-1.75830054
H	2.24352735	-4.07572182	-2.49527505
N	2.32249731	0.04992708	-0.02945494
H	1.31990295	0.02353674	-0.01619544
N	0.05228942	1.40979249	0.42075933
N	0.11575520	-1.41293617	-0.46405919

C	5.34968274	-7.26761387	-1.22677705
O	5.12373264	-7.97656946	-2.18395922
O	6.24704442	-7.53611200	-0.26759362
C	-7.25589047	-3.31807915	1.46451541
O	-7.70422235	-4.20630013	0.77074608
O	-7.84176478	-2.84371056	2.57366251
C	-7.43352702	2.93650293	-1.41333439
O	-7.91740130	3.80324523	-0.71624079
O	-8.00933923	2.42813969	-2.51255790
C	5.10728863	7.48352718	0.89703954
O	5.88558096	7.86248995	0.04762941
O	4.79570165	8.16141234	2.01000131
C	5.46281194	9.43468069	2.17516193
H	5.09097915	9.83412871	3.11755908
H	6.54441187	9.28875536	2.21289250
H	5.21459809	10.09863420	1.34440023
C	-9.30305605	2.97823875	-2.85159158
H	-9.21825709	4.05133644	-3.03617378
H	-9.61277080	2.45163712	-3.75318248
H	-10.00932438	2.80542917	-2.03674541
C	-9.10054262	-3.46112000	2.92792615
H	-8.95781621	-4.52896584	3.10720456
H	-9.42479497	-2.95412212	3.83562917
H	-9.82582798	-3.32260419	2.12331074
C	6.97992078	-8.77359845	-0.42658290
H	7.54284139	-8.75809371	-1.36224136
H	7.65017743	-8.82332989	0.43033081
H	6.28986100	-9.61999036	-0.43223822
H	0.14615852	0.80569540	1.23095290
H	0.17139388	-0.79769831	-1.26933854

Optimised structure of H_2Trip^+

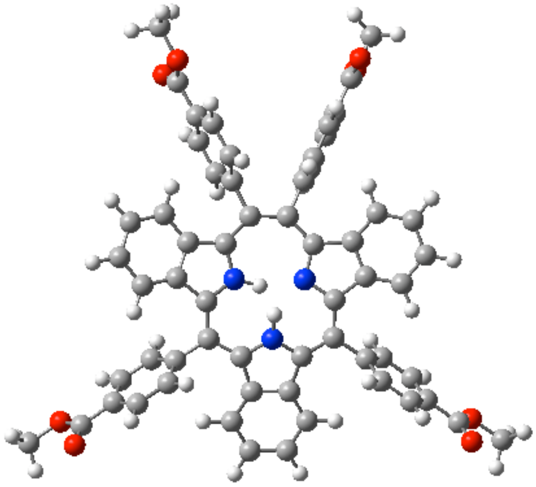

Cartesian coordinates of H_2Trip^+ calculated by DFT at the B3LYP/6-31G(d) level of theory

C	-3.09822400	-1.14177000	-0.47127900
C	-4.39315900	-0.70076300	0.02833900
C	-4.39406500	0.72912500	0.00712300
C	-3.09723200	1.15446600	-0.49998400
C	-2.40403700	2.37680400	-0.48408500
C	-0.97833100	2.41600300	-0.52966000
C	-0.11792700	3.59042100	-0.68371800
C	1.21823600	3.12399000	-0.57477700
C	1.12008600	1.65139200	-0.39428300
C	2.19178900	0.71704300	-0.24120600
C	2.20968500	-0.71770500	-0.20014300
C	1.16525400	-1.67395300	-0.32017600
C	1.21953500	-3.12130300	-0.54474000
C	-0.12693900	-3.58030300	-0.64904400
C	-1.00234900	-2.43808000	-0.45337600
C	-2.41959500	-2.37390000	-0.42527000
C	-5.48844200	-1.40188400	0.55295300
H	-5.49861200	-2.48389700	0.58331300
C	-6.57465300	-0.67602900	1.02645300
H	-7.43681300	-1.20438000	1.42190100
C	-6.57652000	0.73002500	1.00352400
H	-7.44045000	1.26849700	1.38107600
C	-5.49170300	1.44341200	0.50803200
H	-5.50429500	2.52577100	0.50352500
C	-0.39922500	4.93441500	-0.95077700
H	-1.41580600	5.28351500	-1.07285500
C	0.66128700	5.82978300	-1.06635300
H	0.46055100	6.87670300	-1.27317000
C	1.97940900	5.38775900	-0.91545300
H	2.79778200	6.09700300	-0.99685400
C	2.27066500	4.04451900	-0.67611400

H	3.30312400	3.74822300	-0.58402200
C	2.27269400	-4.03721600	-0.71282300
H	3.30523000	-3.73459100	-0.63265400
C	1.97393600	-5.36414100	-0.99633600
H	2.78705600	-6.07224300	-1.12475500
C	0.64659600	-5.80217500	-1.13062200
H	0.44384600	-6.84151000	-1.37049900
C	-0.41033500	-4.91906700	-0.96158200
H	-1.42942700	-5.26305500	-1.07613500
C	-3.22208100	-3.61858300	-0.25519500
C	-3.11545000	-4.38363900	0.91799800
H	-2.43041200	-4.07268200	1.70139300
C	-3.88709900	-5.53026900	1.08762000
H	-3.80743800	-6.11386300	1.99763600
C	-4.77369300	-5.93445000	0.08033600
C	-4.88358200	-5.17663800	-1.09251100
H	-5.57333800	-5.50659300	-1.86225000
C	-4.11953100	-4.02495800	-1.25686300
H	-4.20905600	-3.44036500	-2.16808500
C	3.56191600	-1.35537100	0.01336200
C	4.46716700	-1.54672500	-1.04004400
H	4.20657300	-1.21777300	-2.04108900
C	5.69146000	-2.17337000	-0.81952600
H	6.38547700	-2.32030700	-1.63924700
C	6.03108600	-2.61818100	0.46506900
C	5.13048600	-2.43169300	1.52025900
H	5.40673300	-2.78331700	2.50881200
C	3.90536100	-1.81046600	1.29461900
H	3.20831200	-1.67736700	2.11733500
C	3.55377600	1.35621700	-0.11786300
C	4.34025800	1.59999700	-1.25370300
H	3.96616300	1.32784400	-2.23658600
C	5.58583800	2.20744900	-1.13340100
H	6.20113800	2.40254700	-2.00554800
C	6.06794900	2.58473400	0.12681200
C	5.28700400	2.34655600	1.26421000
H	5.65700300	2.63910000	2.24027700
C	4.03891000	1.73800300	1.13936600
H	3.43610200	1.56057700	2.02550900
C	-3.19974500	3.62450700	-0.30273800
C	-3.10402600	4.36576200	0.88646400
H	-2.42825900	4.03714600	1.67060700
C	-3.87318100	5.51194700	1.06980600
H	-3.80152900	6.07716000	1.99203100
C	-4.74575700	5.94042400	0.06026700
C	-4.84422400	5.20673500	-1.12874700
H	-5.52332700	5.55471500	-1.90003800
C	-4.08360800	4.05449500	-1.30633500
H	-4.16523600	3.48830100	-2.22988200
N	-2.45646600	0.00127700	-0.85522900
H	-1.56725000	0.03353100	-1.33100300
N	-0.16689600	-1.35889400	-0.27418400

N	-0.19717100	1.30588100	-0.38313000
C	-5.59523400	7.16561600	0.19483600
O	-6.36309400	7.55030900	-0.66297500
O	-5.40955100	7.78848900	1.37400100
C	7.41294600	3.23492400	0.19211700
O	8.10533600	3.45478600	-0.78103700
O	7.76862100	3.55048700	1.45283000
C	7.33015300	-3.29535500	0.76642100
O	7.64755300	-3.68851600	1.87050400
O	8.10687100	-3.42045800	-0.32739800
C	-5.62641200	-7.15938600	0.20048800
O	-6.40325300	-7.52503700	-0.65743000
O	-5.43161700	-7.80402100	1.36595700
C	-6.22083400	-8.99576100	1.55343100
H	-5.94073400	-9.37729600	2.53487200
H	-7.28584100	-8.75424000	1.52025700
H	-5.99425100	-9.72689300	0.77378600
C	9.37990100	-4.05898500	-0.11180600
H	9.23687700	-5.07556000	0.26264500
H	9.86726800	-4.07097100	-1.08641400
H	9.97195900	-3.49157900	0.61027700
C	9.05672800	4.17957300	1.59219100
H	9.08473100	5.11709400	1.03159300
H	9.17582700	4.36367100	2.65967800
H	9.84451100	3.51828900	1.22327000
C	-6.19604400	8.97948200	1.57481500
H	-7.26167500	8.74276300	1.52826600
H	-5.92333600	9.34335000	2.56505600
H	-5.96022600	9.72309400	0.80982800
H	-0.41339900	-0.36731300	-0.07797100

Optimised structure of $H_3Trip^{\bullet+}$

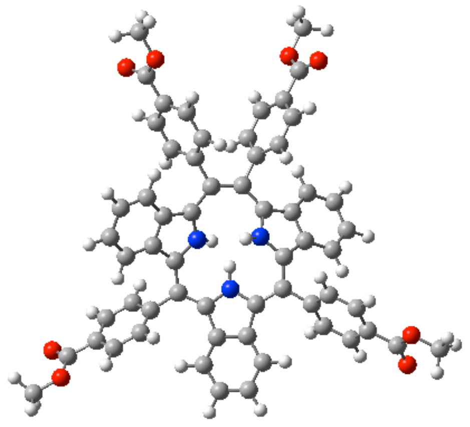

Cartesian coordinates of $H_3Trip^{\bullet+}$ calculated by DFT at the B3LYP/6-31G(d) level of theory

C	3.03608600	1.25457500	0.19546800
C	4.44940400	0.85956600	0.12319600
C	4.50042500	-0.51650500	-0.26271900
C	3.12185800	-1.02328500	-0.30616100
C	2.51742800	-2.27262400	-0.43779300
C	1.09109600	-2.41329200	-0.18035100
C	0.44858500	-3.34687000	0.68577600
C	-0.87841500	-2.86306400	0.95221800
C	-1.06739600	-1.65144400	0.20931900
C	-2.21957400	-0.78144700	0.14079200
C	-2.27580700	0.59256300	-0.16337000
C	-1.19663900	1.55013500	-0.25054200
C	-1.11494100	2.76959700	-1.00034400
C	0.17380500	3.35745900	-0.75638800
C	0.90084000	2.48106600	0.10289800
C	2.33764500	2.45289900	0.33697200
C	5.63312400	1.56831200	0.35132800
H	5.61169700	2.59700100	0.68748200
C	6.85207100	0.93031100	0.13234800
H	7.77613500	1.47335000	0.30574300
C	6.90037500	-0.39378900	-0.32376600
H	7.86118200	-0.86033000	-0.51883900
C	5.73217900	-1.12790200	-0.51704900
H	5.78589200	-2.15467800	-0.85538900
C	0.93019900	-4.50927000	1.33337500
H	1.92658900	-4.88152000	1.13348400
C	0.10165800	-5.16710500	2.21701200
H	0.45080500	-6.06913800	2.71042800
C	-1.19878000	-4.68032200	2.50021500
H	-1.82123500	-5.21420800	3.21204500
C	-1.69021500	-3.54391200	1.89201400
H	-2.68223200	-3.18713900	2.13331800
C	-1.99151700	3.37863900	-1.93126400
H	-2.95586500	2.94344600	-2.15560600

C	-1.59916800	4.54533000	-2.55396800
H	-2.27135800	5.02361400	-3.26007900
C	-0.33652900	5.13371300	-2.29369200
H	-0.06518900	6.05576000	-2.79891500
C	0.55351500	4.54928900	-1.41819700
H	1.52098300	4.99949900	-1.23744300
C	3.01613600	3.76449400	0.51246200
C	2.63862500	4.59745000	1.57973500
H	1.88077300	4.25778900	2.27999500
C	3.23118100	5.84497500	1.75710500
H	2.94089200	6.47462300	2.59036900
C	4.20616800	6.29209900	0.85676300
C	4.57627500	5.47699800	-0.22142400
H	5.31999300	5.84599200	-0.92010700
C	3.98833700	4.22836600	-0.39292500
H	4.26558300	3.61474800	-1.24478500
C	-3.62308800	1.19533800	-0.43539200
C	-4.42350100	0.73926700	-1.49546000
H	-4.07217700	-0.07444800	-2.12211000
C	-5.65784600	1.32674900	-1.75935300
H	-6.26495400	0.97392400	-2.58524700
C	-6.12123700	2.37778600	-0.95680200
C	-5.33053200	2.83672600	0.10438100
H	-5.70458800	3.64978100	0.71761800
C	-4.09163400	2.25844000	0.35506200
H	-3.48067200	2.62849500	1.17365300
C	-3.51093900	-1.48819100	0.43241800
C	-3.90686600	-2.58412500	-0.35287800
H	-3.28211100	-2.90404400	-1.18211500
C	-5.09185500	-3.25895000	-0.08362200
H	-5.40992800	-4.09823400	-0.69312500
C	-5.89906600	-2.86552900	0.99138700
C	-5.50717700	-1.78205900	1.78879500
H	-6.12670600	-1.47925000	2.62524000
C	-4.32774300	-1.09819200	1.50630300
H	-4.03222000	-0.25987900	2.12946200
C	3.29045200	-3.52794700	-0.63187100
C	4.31972000	-3.91571100	0.24488000
H	4.57311300	-3.28161900	1.08918000
C	4.99807600	-5.11765700	0.05870000
H	5.78400500	-5.41289500	0.74470500
C	4.65758100	-5.95957900	-1.00877900
C	3.62610300	-5.58723200	-1.87949900
H	3.37256400	-6.25123200	-2.69913000
C	2.94629000	-4.38932000	-1.68877600
H	2.14553400	-4.10939800	-2.36725400
N	2.35524200	0.08665800	-0.04670200
H	1.35474600	0.04773500	-0.03988900
N	0.03005300	1.43550900	0.39282700
N	0.13643100	-1.43860300	-0.45243100
C	5.34806800	-7.26231400	-1.26160400
O	5.06212800	-8.01112100	-2.17324100

O	6.32160300	-7.50768100	-0.36333700
C	-7.15648200	-3.63655400	1.23689200
O	-7.51572100	-4.57663700	0.55737800
O	-7.84406200	-3.17065700	2.29784500
C	-7.43966700	3.04597500	-1.18335600
O	-7.86337000	3.95407500	-0.49743700
O	-8.10239600	2.52715000	-2.23540300
C	4.87824100	7.62227900	0.98575100
O	5.72076500	8.02913800	0.21176800
O	4.44332900	8.31325800	2.05637100
C	5.05049700	9.60616800	2.24653100
H	4.58980100	10.01325700	3.14621900
H	6.13038300	9.50268200	2.37662100
H	4.85309400	10.24876300	1.38516000
C	-9.38414200	3.12284900	-2.51297600
H	-9.26966800	4.18805000	-2.72795200
H	-9.76849800	2.59179300	-3.38348900
H	-10.05333100	3.00024700	-1.65790800
C	-9.07027300	-3.86641100	2.59294600
H	-8.86808300	-4.91845600	2.80810600
H	-9.48484600	-3.36559300	3.46751700
H	-9.75799900	-3.80014700	1.74642200
C	7.03167700	-8.74826700	-0.54583400
H	7.51808800	-8.76673500	-1.52403400
H	7.77077500	-8.77981200	0.25431300
H	6.34382200	-9.59397500	-0.47166100
H	0.14199700	0.84150300	1.20642600
H	0.18679100	-0.83771000	-1.26717200

Cartesian coordinates of H_3Trip/O_2 calculated by DFT at the B3LYP/6-31G(d) level of theory

C	-2.75507300	1.13162000	-0.29808500
C	-3.99202400	0.68267300	0.34729600
C	-3.96788200	-0.75151500	0.39682100
C	-2.72034300	-1.20170100	-0.21892000
C	-2.02169300	-2.41105400	-0.27338200
C	-0.59559500	-2.39033400	-0.48893800
C	0.25640200	-3.34675900	-1.15040400
C	1.58758700	-2.80797100	-1.12097200
C	1.50561800	-1.52712800	-0.43580300
C	2.60199000	-0.61232000	-0.10810300
C	2.60369000	0.78377300	-0.08683700
C	1.52106400	1.70889000	-0.39636600
C	1.54347900	3.07947600	-0.85027500
C	0.17346900	3.51831200	-0.97214200
C	-0.67594500	2.43657800	-0.55730400
C	-2.10445800	2.36669800	-0.38557200
C	-5.04046300	1.39061600	0.95378100
H	-5.06400800	2.47206200	0.93478300
C	-6.05861700	0.67768100	1.58711400
H	-6.87765400	1.21619600	2.05256700
C	-6.03257800	-0.72860400	1.64011800
H	-6.83109300	-1.26020200	2.14738000
C	-4.99011700	-1.44934700	1.05804400
H	-4.97343100	-2.52967200	1.11888000
C	-0.00938500	-4.55867600	-1.82062700
H	-1.01455200	-4.96071300	-1.85987300
C	1.04103200	-5.23054100	-2.43368300
H	0.85259700	-6.16722000	-2.94905700
C	2.35487100	-4.70818300	-2.39909100
H	3.15778700	-5.25379300	-2.88488800
C	2.63418100	-3.50650800	-1.75751000
H	3.64527400	-3.12336800	-1.74561400
C	2.58540500	3.96302000	-1.21819500
H	3.62032700	3.66831200	-1.12418500
C	2.26822700	5.22366500	-1.70004500
H	3.06676800	5.90377300	-1.97921100
C	0.92125300	5.63980000	-1.84212300
H	0.70660200	6.62742700	-2.23769900
C	-0.12244200	4.80099200	-1.48529300
H	-1.14849900	5.12405300	-1.60435200
C	-2.88223500	3.62549900	-0.22419500
C	-3.96814700	3.91212000	-1.07553300
H	-4.22128500	3.21046100	-1.86400200
C	-4.70812000	5.08871500	-0.91836400
H	-5.53798300	5.29650600	-1.58651700
C	-4.37547600	5.99863600	0.09267200
C	-3.29790300	5.72471300	0.94541700
H	-3.03774500	6.42322300	1.73431000
C	-2.55664100	4.55084800	0.78818600

H	-1.72435700	4.33944400	1.45099500
C	3.85676300	1.48601200	0.39021800
C	3.91162300	1.91743900	1.72740400
H	3.07055900	1.71578100	2.38336500
C	5.04131800	2.58145100	2.21704700
H	5.06973900	2.89997000	3.25430900
C	6.13015900	2.83310700	1.37463100
C	6.08360200	2.41330300	0.04042100
H	6.92371600	2.60405000	-0.62031300
C	4.95513000	1.74407600	-0.44705600
H	4.92199700	1.43083600	-1.48541300
C	3.86000100	-1.34400300	0.30447500
C	3.86377800	-2.01189400	1.54342300
H	2.98322200	-1.95210900	2.17522800
C	4.99343600	-2.72035200	1.96565400
H	4.98303600	-3.22113500	2.92880900
C	6.13075900	-2.78604900	1.15248800
C	6.13361500	-2.13303900	-0.08526900
H	7.00986800	-2.18090600	-0.72464800
C	5.00779900	-1.41546300	-0.50405600
H	5.01473700	-0.92083400	-1.46916500
C	-2.72385500	-3.70727600	-0.10811000
C	-3.89149500	-4.00013700	-0.84304000
H	-4.27146100	-3.26671200	-1.54704200
C	-4.54737000	-5.22448700	-0.68391900
H	-5.44083100	-5.43620100	-1.26291900
C	-4.04784000	-6.17897300	0.21105600
C	-2.88674700	-5.90160300	0.94459100
H	-2.49648100	-6.63523900	1.64267900
C	-2.22858400	-4.68005500	0.78513500
H	-1.33267400	-4.46442700	1.35718400
N	-2.11882000	-0.03587100	-0.67744400
H	-1.29504200	-0.06358200	-1.25380300
N	0.18914900	1.39085400	-0.25559700
N	0.19856500	-1.31554500	-0.07185200
H	7.00640100	3.34936800	1.75389400
H	-4.94863500	6.91218500	0.21440200
H	-4.55611700	-7.13006800	0.33361200
H	7.00527200	-3.34025600	1.47892700
H	-0.07629800	-0.97645700	1.50861500
O	-0.25308400	-0.75736400	2.50771000
O	0.97855400	-0.83272000	3.14390400
H	-0.06711200	0.45939700	0.08941000
